# Resting-State fMRI in Chronic Patients with Disorders of Consciousness: The Role of Lower-Order Networks for Clinical Assessment

**DOI:** 10.3390/brainsci12030355

**Published:** 2022-03-07

**Authors:** Jean Paul Medina, Anna Nigri, Mario Stanziano, Ludovico D’Incerti, Davide Sattin, Stefania Ferraro, Davide Rossi Sebastiano, Chiara Pinardi, Giorgio Marotta, Matilde Leonardi, Maria Grazia Bruzzone, Cristina Rosazza

**Affiliations:** 1 Neuroradiology Unit, Diagnostic and Technology Department, Fondazione IRCCS Istituto Neurologico Carlo Besta, 20133 Milan, Italy; jeanpaul.medina@istituto-besta.it (J.P.M.); mario.stanziano@istituto-besta.it (M.S.); ludovico.dincerti@meyer.it (L.D.); stefania@uestc.edu.cn (S.F.); chiara.pinardi@asst-nordmilano.it (C.P.); maria.bruzzone@istituto-besta.it (M.G.B.); 2 Neurosciences Department “Rita Levi Montalcini”, University of Turin, 10126 Turin, Italy; 3 Neuroradiology Unit, Children’s Hospital A. Meyer—University of Florence, 50139 Florence, Italy; 4 IRCCS Istituti Clinici Scientifici Maugeri di Milano, 20138 Milan, Italy; davide.sattin@icsmaugeri.it; 5 MOE Key Laboratory for Neuroinformation, School of Life Science and Technology, University of Electronic Science and Technology of China, Chengdu 611731, China; 6 Epileptology Unit, Department of Neurophysiology and Diagnostic, Fondazione IRCCS Istituto Neurologico Carlo Besta, 20133 Milan, Italy; davide.rossi@istituto-besta.it; 7 Medical Physics Unit, Asst Nord Milano, Sesto San Giovanni, 20099 Milan, Italy; 8 Department of Nuclear Medicine, Fondazione IRCCS Ca’ Granda Ospedale Maggiore Policlinico, 20122 Milan, Italy; giorgiomarotta58@gmail.com; 9 Neurology, Public Health, Disability Unit, Fondazione IRCCS Istituto Neurologico Carlo Besta, 20133 Milan, Italy; matilde.leonardi@istituto-besta.it; 10 Department of Humanistic Studies, University of Urbino Carlo Bo, 61029 Urbino, Italy

**Keywords:** disorders of consciousness, resting-state fMRI, visual, auditory and sensorimotor networks

## Abstract

Resting-state fMRI (rs-fMRI) is a widely used technique to investigate the residual brain functions of patients with Disorders of Consciousness (DoC). Nonetheless, it is unclear how the networks that are more associated with primary functions, such as the sensory–motor, medial/lateral visual and auditory networks, contribute to clinical assessment. In this study, we examined the rs-fMRI lower-order networks alongside their structural MRI data to clarify the corresponding association with clinical assessment. We studied 109 chronic patients with DoC and emerged from DoC with structural MRI and rs-fMRI: 65 in vegetative state/unresponsive wakefulness state (VS/UWS), 34 in minimally conscious state (MCS) and 10 with severe disability. rs-fMRI data were analyzed with independent component analyses and seed-based analyses, in relation to structural MRI and clinical data. The results showed that VS/UWS had fewer networks than MCS patients and the rs-fMRI activity in each network was decreased. Visual networks were correlated to the clinical status, and in cases where no clinical response occurred, rs-fMRI indicated distinctive networks conveying information in a similar way to other techniques. The information provided by single networks was limited, whereas the four networks together yielded better classification results, particularly when the model included rs-fMRI and structural MRI data (AUC = 0.80). Both quantitative and qualitative rs-fMRI analyses yielded converging results; vascular etiology might confound the results, and disease duration generally reduced the number of networks observed. The lower-order rs-fMRI networks could be used clinically to support and corroborate visual function assessments in DoC.

## 1. Introduction

Disorders of Consciousness (DoC) include a spectrum of conditions ranging from the coma, vegetative state/unresponsive wakefulness state (VS/UWS) to a minimally conscious state (MCS), which results from alterations in arousal and/or awareness caused by severe brain damage.

While some patients recover consciousness, others survive in a chronic VS/UWS or MCS condition, with MCS having a better prognosis than VS/UWS [1,2] and the clinical outcome is better for traumatic brain injury than vascular and anoxic etiologies [3,4,5].

The clinical examination based on standardized behavioral scales remains the gold standard for the diagnosis of DoC [6], although two recent guidelines, the American Academy of Neurology [7] and the European Academy of Neurology (EAN) [8], emphasize the role of neuroradiological findings to improve diagnostic accuracy and classification between VS/UWS and MCS. In fact, neuroimaging techniques represent a crucial part of the assessment, providing information about the structural and functional integrity of the patient’s brain [9,10,11]. In particular, resting-state functional MRI (rs-fMRI) is applied to the study of DoC to detect residual brain activity. Emerging evidence leads us to consider the usefulness of rs-fMRI in addition to MRI examination as part of a multimodal assessment, as also recommended in the 2020 EAN guideline [8].

The rs-fMRI technique measures the spontaneous neuronal activity that is generated by the brain at rest, reflected by low-frequency fluctuations in the BOLD signal [12]. Regions that show strong synchrony over time are functionally connected and form separate rs-fMRI networks [13]. Several neural networks have been identified and broadly classified into two groups [14]: higher-order networks such as the default mode network (DMN) associated with introspective processes and self-awareness [15], and lower-order networks such as the sensorimotor (SM), visual, and auditory networks (AUD), which exhibit patterns of activity that resemble activities observed during tasks, and are associated with more specialized functions. The SM network, for example, involves regions engaged in sensorimotor functions [16], and its activity at rest displays a degree of hemispheric lateralization that correlates with that one observed during a finger-tapping task [17]. The visual network can be divided into lateral (LVIS) and medial (MVIS) networks, with the first including mesial visual areas, and the latter including lateral occipito-temporal regions. The AUD network consists primarily of Heschl’s gyrus and the superior temporal gyrus. In addition, functional connectivity observed at rest in healthy subjects is influenced by experience and can be modulated by prior history of co-activation during active behavior [18,19,20,21]; therefore, functional connectivity in patients with DoC can provide information on the residual functioning of sensory modalities such as the visual, auditory, and somatosensory systems.

In fact, rs-fMRI has been applied to DoC to assess residual neurofunctional activity. The DMN activity was shown to be absent in brain death and generally stronger in MCS than in VS/UWS [22,23,24]. Beyond the well-studied DMN, other rs-fMRI networks might be able to define consciousness, although the number of studies is limited. Among the networks associated with primary functions (SM, LVIS, MVIS, AUD), one study examined 51 cases (26 MCS, 19 VS/UWS and 5 in coma) generally in the subacute phase and validated the classification on another 22 patients with DoC [25]. Each network could discriminate MCS from VS/UWS with high accuracy (>80%), with the AUD having the best performance. Another study involving 16 patients with DoC in the subacute phase also revealed impairment in rs-fMRI connectivity in the SM and AUD networks, but not in the VIS network relative to healthy controls [14]. Lastly, functional connectivity has been studied both within and between networks: rs-fMRI measurements were associated with 1-year clinical outcome in comatose patients [26] and could be used, along with clinical data, to predict prognosis in the chronic phase [27]. Although rs-fMRI can be useful as a supplement to structural MRI as part of a multimodal assessment in DoC [8,28], a well-preserved map of the rs-fMRI network does not provide evidence of consciousness and the difference between VS/UWS and MCS can be undetected [23,29]. In this context, structural MRI combined with rating scales of anatomical damage has been less investigated in the study of DoC, although recent evidence shows that this type of MRI-based assessment can be useful in discriminating VS/UWS from MCS [24,30].

In regard to the differences in the etiology, a recent multicentric study has shown that post-anoxic patients have the highest clinical complexity, as they have a lower level of consciousness, higher functional disabilities and a greater need for medical devices. This is followed by vascular patients who demonstrate a greater premorbid clinical comorbidity and then by traumatic patients who demonstrate a smaller clinical complexity [31].

However, there is a lack of characterization of these lower-order rs-fMRI networks in terms of their occurrence, etiology, and relationship to behavioral responses. Further, the diagnostic accuracy of networks between VS/UWS and MCS needs to be verified in a larger sample.

In this study, we investigated the integrity of the 4 lower-order rs-fMRI networks (SM, LVIS, MVIS, AUD) associated with primary functions in 109 chronic patients with DoC and emerged from DoC with different etiologies and on average a disease duration > 2 years.

The aim is to better characterize patients in terms of low-order rs-fMRI networks and explore patients’ brain residual functional activity in relation to the clinical data, i.e., etiology, disease duration and CRS-R subscores. In addition, the 4 rs-fMRI networks were evaluated alongside their structural MRI, as occurs in clinical practice, and analyzed (i) individually and (ii) by combining the information of the four networks together. We used seed-based analysis (SBA) with map selection among different seeds [32] and independent component analysis (ICA) with the integration of three pieces of information (spatial maps, time series and power spectra) [33,34], developing a qualitative rs-fMRI rating and a quantitative rs-fMRI map intensity index.

## 2. Subjects and Methods

### 2.1. Participants

A group of 122 adult patients with DoC and emerged from DoC in chronic phase, admitted to a 1-week program of multidisciplinary assessment at the Coma Research Center (CRC) of the Fondazione IRCCS Istituto Neurologico “Carlo Besta”, Milan, Italy, was enrolled. Thirteen patients were excluded for excessive head movement during the MRI session (3) or excessive artefactual MRI data (10). Thus, according to the Aspen criteria, the study included 65 patients in VS/UWS, 34 in MCS and 10 with severe disability (SD) emerged from MCS, as assessed with the CRS-R. Etiology included 33 traumatic brain injury, 39 vascular brain injury and 37 anoxic brain injury. Median disease duration was 27 months (range 2–252, >12 months for 82 cases), and the median age was 50 years (range 19–83). Demographic and clinical characteristics are reported in Table 1.

Patients were clinically assessed with the Italian version of the Coma Recovery Scale-Revised CRS-R) [35,36] and with the CRS-R—Modified score [37]; each patient was independently assessed 4 times by experienced raters and the best response was used to establish the final score. Patients also underwent a multimodal assessment comprising evoked potentials (EPs) to assess the presence/absence of the visual, auditory and sensory pathways (as reported in [38]) and FDG-PET, to have a measure of metabolism (as reported in [24,39,40]).

A total of 34 healthy participants (median age 39 years, range 17–66) with no history of neurological deficits were recruited as controls. The study was approved by the ethics committee of the “Carlo Besta” Institute. Written informed consent was obtained from the legally authorized representative of the patients and healthy participants.

### 2.2. MRI Data Acquisition

Scanning was performed on a 3T scanner equipped with a 32-channel head coil (Achieva TX; Philips Healthcare, Best, Amsterdam, The Netherlands). For rs-fMRI data, gradient echo-planar images (EPI) were acquired (repetition time = 2.8 s, echo time = 30 milliseconds, flip angle = 70°, 2.5 mm isotropic voxel size, 90 × 95 matrix size, 50 slices with 10% gap, ascending order, 200 volumes); the sequence duration was ~9.5 min. Sagittal 3D T1-weighted, 2D T2, T2* and FLAIR-weighted images were also acquired. When patient posture allowed, the head was restrained using foam pillows, and a knee wedge was positioned to minimize spine movement and discomfort. Sedation was never performed.

### 2.3. rs-fMRI Data Preprocessing

Data were preprocessed using SPM12, FSL, and in-house code running under Matlab (MathWorks, Natick, MA, USA) [41]. Preprocessing consisted of rigid-body realignment, slice time correction and identification of outlier scans for the scrubbing procedure using a framewise displacement greater than 2 mm (see [25] for similar choices) in FSL volume outlier function. The 3D T1-weighted image was co-registered to mean EPI image, and tissue probability maps of white matter (WM), cerebrospinal fluid (CSF) and grey matter (GM) were extracted.

Multiple linear regressions were used to remove additional confounds, including the 6 head movement parameters, mean WM and CSF signals, as well as linear trend obtained with the 4rd-order polynomial and outlier volumes. A 0.01–0.1 Hz band-pass filtering was applied. The 3D T1 and EPI images were normalized to MNI template. EPI images were spatially smoothed using 6 mm fullwidth at half-maximum Gaussian filter.

### 2.4. Independent Component Analysis (ICA)

ICA was performed using MELODIC (FSL tool https://fsl.fmrib.ox.ac.uk/fsl/fslwiki/MELODIC, accessed on 31 December 2021) with a fixed number of 30 components [42], which were thresholded at z ≥ 3. Group ICA maps were computed over healthy subjects, and binary templates of the 4 networks were created. Cross-correlation between template and patient single component was computed for each network with fslcc to obtain a similarity index and have a preliminary selection of the more likely neural networks. Then, the component corresponding to each network was identified upon agreement of two experienced observers who considered the spatial map, time series shape, and power spectral density [34]. A component was deemed a candidate network if it exhibited focal activity in the network nodes and specificity to the rest of the brain [43], if the power spectrum presented low frequency only, and if the time series was smooth. Melodic outputs were resampled with a 2 mm voxel.

### 2.5. Seed-Based Analysis (SBA)

For each patient, SBA was performed with DPARSF-A [44] using a 6 mm radius sphere for each node of the 4 networks. A total of 10 seeds was defined, where SM had 4 seeds, 2 located in the left and right pre- and post-central gyri and 2 in left and right supplementary motor area (SMA); AUD had 2 seeds located in the left and right Heschl’s gyrus; LVIS had 2 seeds located in left and right inferior occipital and fusiform gyrus; and, finally, MVIS had 2 seeds located in the left and right lingual gyrus and calcarine cortex. The mean time-course of each seed was entered into a whole-brain correlation analysis, and corresponding statistical maps were thresholded at z > 0.55.

In order to select the best seed-based maps, two methods were used to select the location of the seed: the first one was based on the peak coordinates taken from the literature [25,27,45], while the second one was based on subject-specific coordinates placed on the most preserved GM area visible on T1 scan. For each node, the two maps were compared and the less noisy and more specific one was chosen [32,46]. To assess the contribution of each hemisphere and avoid overestimation of connectivity maps, left and right seeds were analyzed separately [47].

### 2.6. rs-fMRI Rating (Qualitative Analysis)

For each patient, a rating was performed by two expert investigators blind to patient data. Each node of ICA and SBA maps was assessed according to the following scale: 0 (no map or undefined map), 1 (map with low specificity and ambiguous cluster), 2 (neuronal map with specific cluster on a few slices) and 3 (neuronal map with highly specific cluster on many slices). For SBA maps the threshold was changed to reduce noise when necessary. In case of large disagreement between the two raters, the scores were reconsidered. The scores obtained by two raters were averaged together. Inter-rater agreement was *p* = 0.79, indicating good reliability [48].

The presence of a network was defined as a score ≥ 2 on at least one node of ICA or SBA for MVIS, LVIS and AUD networks. For the detection of the SM network, a score ≥ 2 on at least two nodes of ICA or SBA maps was necessary. For each diagnostic group, the number of networks observed was calculated.

### 2.7. rs-fMRI Map Intensity (Quantitative Analysis)

In order to have a quantitative analysis in addition to the rs-fMRI rating, the mean intensity of ICA and SBA maps was extracted for each node, added together and included: (i) as two predictors (left and right hemisphere) in the multivariate logistic regression analysis; (ii) as single variable per network with the Mann–Whitney U test.

### 2.8. Voxelwise Group Map Analyses

Group-level analyses were performed using Statistical NonParametric Mapping (http://www.nisox.org/Software/SnPM13/, accessed on 31 December 2021) for each network. This was chosen as data were not normally distributed due to the presence of zero-filled maps. The analysis was conducted on SBA maps only because ICA provided fewer recognizable networks. The left and right maps were combined considering for each voxel the maximum value [24]. A correlation analysis was performed between each network and its corresponding CRS-R subscale. Maps with rs-fMRI ratings = 0 or 0.5 were not included to avoid spurious correlations.

### 2.9. MRI Rating

Two expert neuroradiologists blind to all patient data rated independently the severity of gross anatomical and signal abnormality of the regions corresponding to the network nodes, according to a 4-score scale ranging from 0 (severely damaged, i.e., parenchyma obliterated and/or intense, pervasive hyperintensity) to 4 (normal-appearing) (see [24]). Ratings were reconsidered in cases of large disagreement; the scores of each node were averaged between raters and then summed for each network. Inter-rater agreement was *p* = 0.77, indicating good reliability [48].

### 2.10. Statistical Analyses

Statistical analyses were performed using R software version 4.0.3 (https://cran.r-project.org/, accessed on 31 December 2021) [49]. The Mann–Whitney U test was used to test VS/UWS vs. MCS differences. Spearman ρ was used to study the correlation of imaging data with clinical data, i.e., with CRS-R total score and the CRS-R subscale scores [36], with the CRS-R—Modified score [37] and disease duration. As regards the correlation with CRS-R subscales, the auditory, visual and motor function subscales were selected as they may most correspond to the low-order rs-fMRI networks.

A multivariate logistic regression model was performed to assess the ability to classify VS/UWS vs. MCS patients for single networks and the 4 networks together. The following 5 imaging models were created, with ICA and SBA scores considered separately for rs-fMRI and using 2 scores per node: (1) rs-fMRI rating (2 scores per node); (2) rs-fMRI map intensity (2 scores per node); (3) MRI rating (1 score per node); (4) rs-fMRI rating and MRI rating (3 scores per node); and (5) rs-fMRI map intensity and MRI rating (3 scores per node). Moreover, clinical variables (etiology, disease duration and age) were added in each of the previous models to create imaging + clinical variable models. A model with only clinical variables was used as a baseline to compare all previous models.

The least absolute shrinkage and selection operator (LASSO) method was used to reduce the dimension of explanatory variables of the logistic regression and make the model easier to interpret. Leave-one-out cross-validation (LOOCV) and 10-fold cross-validation (CV) were used to internally validate the selected model; the results of the 10-fold CV are reported in the Supplementary Material. Accuracy and balanced accuracy obtained after LOOCV were extracted to measure the diagnostic discrimination, together with the estimate of the area under a receiver operating characteristics curve (AUC). The sensitivity and specificity of each AUC were calculated with corresponding CI using the bootstrap method with 2000 stratified replicates. Results are reported in the Supplementary Tables. Following the STARD guidelines [50], false positive and false negative patients were also identified, and diagnostic accuracy was considered as very good for 0.8 < AUC < 0.9, good for 0.7 < AUC < 0.8, sufficient for 0.6 < AUC < 0.7 and bad for 0.5 < AUC <0.6 [51]. The McNemar test was used to compare different logistic regression models for the 4-network assessment.

## 3. Results

### 3.1. Differences between Diagnostic Groups

**Figure 1:**
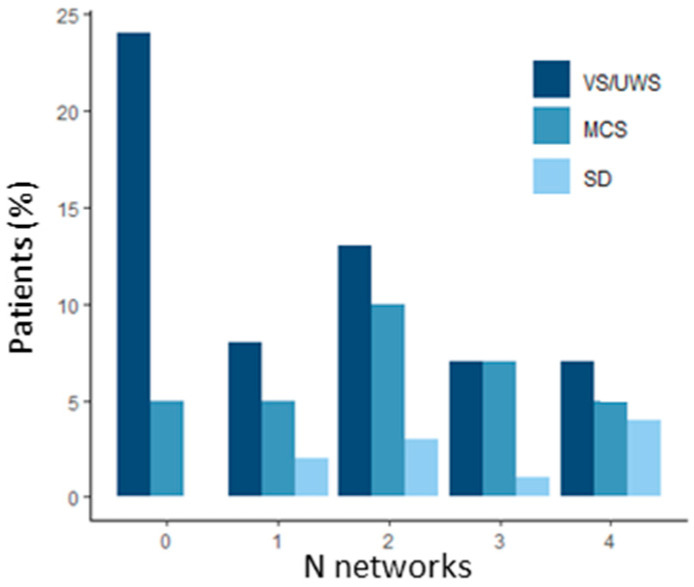
Percentage of patients having 0, 1, 2, 3 or 4 networks for each diagnostic group. Network detection was defined as a score ≥ 2 on at least one node of ICA or SBA maps for LVIS, MVIS, and AUD networks, and defined as a score ≥ 2 on at least two nodes of ICA or SBA maps for the SM Network.

Considering the number of networks observed in each diagnostic group, the most frequent values were 0 for VS/UWS, 2 for MCS and 4 for SD (Figure 1). Each diagnostic group had a range of variation between 0 and 4 except for SD which never had 0.

**Figure 2:**
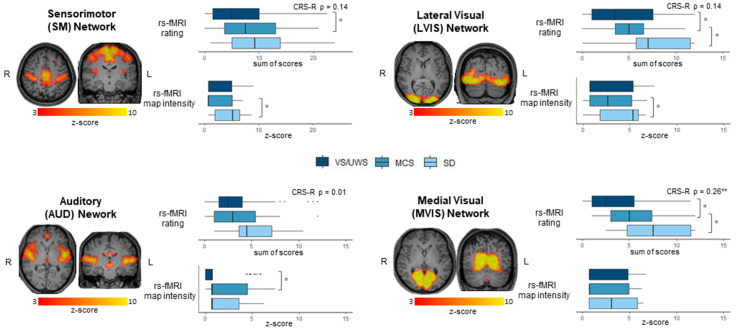
The 4 rs-fMRI networks: sensorimotor (SM), auditory (AUD), lateral visual (LVIS) and medial visual (MVIS) networks. For each network, on the left, the spatial map generated from the group-level ICA on control subjects; on the right, group differences measured with rs-fMRI rating and rs-fMRI map intensity between VS/UWS, MCS and SD patients. Boxplot with medians and interquartile range are reported, with Mann–Whitney Z scores. For each network the correlation of rs-fMRI rating with CRS-R total score is reported. * *p* < 0.05; ** *p* < 0.01.

For each network, the Mann–Whitney test showed a significant difference between VS/UWS and MCS (*p* < 0.05), which was detected more frequently with the rs-fMRI rating than rs-fMRI map intensity (Figure 2). Correlation with CRS-R total score was significant only with the rs-fMRI rating for the MVIS network (ρ = 0.26 *p* < 0.01). Considering only VS/UWS and MCS, the correlation was not significant with CRS-R total score, but was significant with CRS-R -Modified score (ρ = 0.22 *p* < 0.05). Correlation with disease duration was never significant.

### 3.2. Diagnostic Accuracy of Single Networks

Considering the networks individually, logistic regression after applying LASSO showed averaged accuracy around 0.68 with AUC = 0.55 for the rs-fMRI rating and rs-fMRI map intensity models, while balanced accuracy dropped to 0.57 (Table 2, Supplementary Tables S1 and S2). Generally, there were no differences between the two rs-fMRI models. Combining rs-fMRI with MRI data, accuracy scores improved (on average balanced accuracy = 0.58 and AUC = 0.67) albeit not significantly, and adding clinical variables the results improved slightly further (on average balanced accuracy = 0.64 and AUC = 0.74). The model with only clinical variables (etiology, disease duration and age) has accuracy = 0.72, balance accuracy = 0.64 and AUC = 0.71. Henceforth balanced accuracy is used instead of accuracy because it is considered more appropriate for the presence of unbalanced groups. Finally, there were no hemispherical differences in the number of variables selected by LASSO (see Supplementary Table S3 for the description of the variables).

### 3.3. Correspondence between Single Networks and CRS-R Subscales

As reported in Table 3, the scores of the CRS-R subscales were centered on score 2 for the motor function and between scores 1 and 2 for the auditory function: this polarization reduced the possibility of observing significant correlations with rs-fMRI. In contrast, the scores of the visual function were more distributed: the visual function was positively correlated with the rs-fMRI rating of the LVIS (ρ = 0.27, *p* < 0.01) and MVIS (ρ = 0.38, *p* < 0.001) networks, indicating that the higher the visual score, the more recognizable is the rs-fMRI visual map.

**Figure 3:**
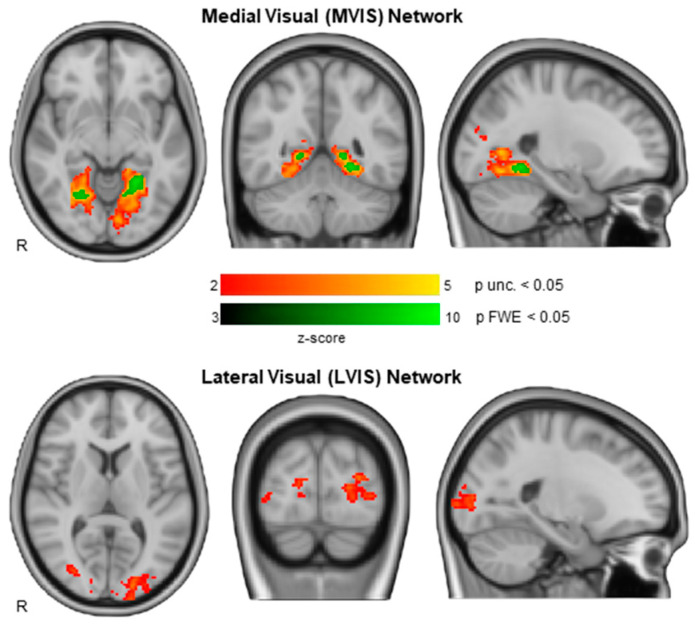
The integrity of the medial visual (MVIS) and lateral visual (LVIS) networks, measured with SBA, correlates with the complexity of the visual response and therefore with the level of consciousness assessed with the CRS-R visual subscale. Results of the nonparametric correlation are thresholded voxelwise at *p* uncorrected <0.05 (in red) and p family-wise error (FWE) *p* < 0.05 (in green).

A voxelwise group analysis performed between the visual networks (LVIS and MVIS) measured with SBA (which included more maps than ICA) and the CRS-R visual function subscale showed significant results. In particular, for the MVIS network on a total of 88 patients (44 VS/UWS, 34 MCS and 10 SD) results displayed the involvement of the lingual gyrus and calcarine cortex, bilaterally at two thresholds, while for the LVIS network on a total of 84 patients (55 VS/UWS, 23 MCS and 6 SD) results displayed the involvement of the inferior occipital areas more on the left side (Figure 3).

Analyzing the correspondence between the CRS-R subscale and network recognizability, we expected an absence of networks when the CRS-R score was zero. However, some networks were detected even when patients had 0 at the subscale: three networks for AUD, six for LVIS and four for MVIS, where the four patients with MVIS had also LVIS (Table 4). As a result, a total of nine patients had a score ≥ 2 for rs-fMRI rating, indicating high network preservation even if the CRS-R subscale score was 0. Since our patients underwent also visual, auditory and somatosensory potentials—and for some cases also FDG-PET—we compared the results of these techniques in terms of presence/absence of EP and FDG-PET hypometabolism with rs-fMRI data: for seven out of nine cases, the EPs showed the integrity of the pathways and FDG-PET showed active areas in agreement with rs-fMRI results. Thus, concordance was reached for seven out of nine cases, indicating that rs-fMRI data were confirmed by EPs or FDG-PET or both.

### 3.4. Diagnostic Accuracy of the 4 Networks

Considering the 4 networks, logistic regression after applying LASSO showed averaged balanced accuracy of 0.65 and 0.67 for the two rs-fMRI models (Table 5, Supplementary Tables S4 and S5).

Combining rs-fMRI with MRI data, accuracy and balanced accuracy did not change.

Adding clinical variables, accuracy slightly improved to 0.82 for rs-fMRI rating + MRI rating (with balanced accuracy = 0.78) and remained at 0.74 for rs-fMRI map intensity + MRI rating (with balanced accuracy = 0.71). Only the latter model (rs-fMRI map intensity + MRI rating + clinical variables) resulted to be significantly different from the model with only clinical variables (with balanced accuracy = 0.64 and AUC = 0.71) in terms of classification results (McNemar test, *p* < 0.05).

Statistically, accuracy and balanced accuracy had near value because the sensitivity and specificity of AUC were similar. Considering the significant variables included by LASSO method there were no significant differences between right and left hemispheres (Table 5 and Supplementary Table S6).

### 3.5. Differences in Etiology

Considering possible differences in etiology, we again conducted logistic regressions analyses after applying the LASSO method (Table 6, Supplementary Tables S7 and S8). In traumatic patients, the LASSO method did not retain any variable. In vascular patients, the balanced accuracy was 0.74 for both rs-fMRI indices. Combining rs-fMRI indices with MRI rating, the results did not change; adding the clinical variables, the accuracy did not improve and only the disease duration was significant as a variable. In post-anoxic patients, the balanced accuracy was 0.63 for rs-fMRI rating and 0.90 for rs-fMRI map intensity. Combining the two rs-fMRI indices with MRI rating, the balanced accuracy became 0.63 and 0.83, respectively. Adding the clinical variables, the balanced accuracy did not change considerably and only the disease duration was significant as a clinical variable (see Supplementary Table S9). Only for this class, LASSO included more variables of the left than the right hemisphere.

Overall, considering the three etiological classes, all networks contributed similarly to accuracy.

Considering the number of networks identified, a positive relation was observed with the CRS-R total score for the total of patients (ρ = 0.26, *p* < 0.01), indicating that as the CRS-R total score increases, the number of networks also increases (Table 7); this relation was stronger for the anoxic class (ρ = 0.39, *p* < 0.05). Generally, the two visual networks were the most frequently recognizable components.

In particular, for traumatic patients, the number of networks identified generally increased as the diagnostic group passed from VS/UWS to MCS to SD (Table 7). The visual networks better distinguished VS/UWS from MCS compared to the other components. For vascular patients the recognition of networks did not increase with diagnosis, rather the effect was opposite, with a higher number of networks for VS/UWS than MCS. Only the AUD network was more frequently observed for MCS than VS/UWS. This might be due to the lesion site, often involving the territory of the middle cerebral artery in vascular injury, with a more severe impact for VS/UWS than MCS. For anoxic patients, the recognition of networks clearly increased as the diagnosis improved; the visual networks were the less recognizable component for VS/UWS (13%), but the more recognizable for MCS (80%).

Finally, the Spearman correlation between the number of networks observed for VS/UWS and MCS (without SD) and CRS-R total score was not significant, nor was the one with CRS-R—Modified score.

Furthermore, there was a negative relation between the number of networks identified and disease duration for the total of patients (ρ = −0.19, *p* < 0.05), indicating that as disease duration increases, the number of networks decreases (Table 7).

Clinical data of all patients are reported in Supplementary Table S10.

## 4. Discussion

In 109 patients with DoC and emerged from DoC we investigated the integrity of the four rs-fMRI networks more related to primary functions, namely the SM, LVIS, MVIS and AUD components, and analyzed data in relation to structural MRI and clinical information (etiology, disease duration and age), as occurs in clinical practice.

### 4.1. Assessment of Single Networks

Our data show that: (i) VS/UWS patients present a lower number of networks compared to MCS patients; (ii) functional connectivity is useful to differentiate VS/UWS from MCS; and (iii) the MVIS and LVIS networks are correlated with the clinical status measured with CRS-R.

In particular, VS/UWS patients were most frequently associated with 0 networks and MCS patients to 2 networks, although both classes ranged from 0 to 4 components. Only SD patients never had any networks and were most frequently associated with 4 components.

The diagnostic accuracy provided by single networks is generally limited when the rs-fMRI data is taken alone (on average balanced accuracy = 0.57 and AUC = 0.55). To represent the clinical reality where rs-fMRI is typically assessed with structural imaging, we included a model with rs-fMRI+MRI data from each network, which however showed low accuracy values (on average a balanced accuracy of 0.58 and AUC = 0.67). As these are lower-order rs-fMRI networks corresponding to primary functions, an inferior diagnostic power is not unexpected compared to higher-order networks such as DMN.

Results are lower than those reported by Demertzi et al. (2015) [25] which demonstrated that visual, auditory and sensorimotor networks assessed individually had a discriminative capacity greater than 80%; however, they studied a smaller sample of patients in a subacute phase and used different statistical methods. To date, no other studies have investigated these networks with respect to the VS/UWS vs. MCS classification and differentiation.

The two rs-fMRI measures used here are rs-fMRI rating and rs-fMRI map intensity: the first one is based on a qualitative, time-consuming assessment indicating the degree of network recognition, probably leading to more interpretable results; the second one results from an automated analysis procedure indicating the mean intensity of the network. Interestingly, both measures yielded similar results, which demonstrates that implementing rs-fMRI into the clinical routine based on expert assessment can be feasible [24], although automatic rating can be quicker especially when the sample size is large.

A potentially relevant question for the clinical assessment refers to the correspondence between the rs-fMRI network and the CRS-R subscale and is whether the presence of a visual, auditory or sensorimotor map can be clinically useful for evaluating patients’ perceptual functioning. In our sample, only the visual subscale could be correlated with the rs-fMRI data, as scores of the motor and auditory CRS-R subscales were centered on a few values. Results showed that the highest the visual score was, the more recognizable resulted the rs-fMRI visual map, indicating that the type of response and the level of behavioral complexity in the visual domain were correlated with the integrity of LVIS and MVIS networks. The voxel-wise analysis also showed a significant correlation between the integrity of the visual networks and the CRS-R visual score: as visual scores > 2 are associated with MCS, a well-preserved rs-fMRI visual map might be associated with a high level of consciousness. This, however, does not imply that the presence of the visual network is a prerequisite for, or a direct sign of consciousness; these correlative results only suggest that the visual networks can provide additional information on the visual functioning in DoC and might be functionally relevant for the state of consciousness of the patient.

More generally, the correspondence between CRS-R subscales and network recognizability allowed us to assess those cases where the clinical score was zero, i.e., no behavioral response, but the rs-fMRI map was present. Obviously, they can be cases of possible overestimation, where a network is erroneously identified, despite a low clinical score; alternatively, they can represent a concrete demonstration of how functional imaging measurements can reveal the presence of residual brain functioning, despite scarce auditory/sensorimotor/visual/cognitive performance. For some cases reported in the literature, the presence of a rs-fMRI network map indicated residual brain function, not detectable at the patient’s bed and associated with clinical recovery [24,52]. In our study, 7 cases had a clinical score = 0 but showed a rs-fMRI network that was clearly recognizable, which was confirmed by EPs and FDG-PET. This finding confirms that the presence of clearly identifiable rs-fMRI networks in patients with DoC may warn clinicians about possible clinical assessment biases and prompt a more careful evaluation of the patient’s condition. Indeed, in some patients, motor impairments can cause difficulties in clinical assessment [53]. In this context, the rs-fMRI technique is a valuable supplementary assessment [8] that can be added to multimodal DoC evaluations, providing information on residual cortical functions.

### 4.2. Assessment of Four Networks

Our data show a significant relationship between the number of networks identified and the clinical status measured with the CRS-R total score (ρ = 0.26, *p* < 0.01). Combining data from the four networks, rs-fMRI map intensity achieved the highest accuracy scores (balanced accuracy = 0.67 and AUC = 0.77) which were even higher when the model included also MRI, yielding very good classification results (balanced accuracy = 0.71 and AUC = 0.80) [51]. Importantly, the model with rs-fMRI map intensity + MRI + clinical variables was significantly better than the one considering only clinical variables. Generally, all networks contributed to the results. We, therefore, advise that rs-fMRI and structural imaging should be considered together to attain the best diagnostic accuracy.

### 4.3. Effect of Etiology and Disease Duration

Two issues, scarcely investigated yet, are the effects of etiology and disease duration in patients with DoC. The ability to classify VS/UWS vs. MCS was assessed separately for the traumatic, vascular and anoxic etiologies. Balanced accuracy and AUC were higher for anoxic and lower for traumatic etiology, while for the vascular class, more networks were apparently observed for VS/UWS than MCS. These results confirm that the VS/UWS vs. MCS distinction is more difficult for the vascular (ischemic and hemorrhagic) etiology, where the damage to cortex and white matter is typically more nuanced and heterogeneous, and clusters of correlated activity are more difficult to judge as neural or artifactual [24,39]. For the anoxic class, the rs-fMRI map intensity had a balanced accuracy of 0.90 and AUC = 0.98, indicating certain ease of classification for this etiology despite the high clinical complexity [31]; the result was confirmed by a significant relationship between the number of networks observed and CRS-R total score (ρ = 0.39, *p* < 0.01). For traumatic patients, there was no discrimination between VS/UWS vs. MCS patients, probably due to the location of the damage which, on average, made accuracy more difficult. Overall, results warn about the impact of etiology on the analysis of rs-fMRI networks where vascular patients are more difficult to classify and suggest subdividing patients by etiology if the patient sample allows it.

Among the four networks, the visual components were the most frequently recognizable in particular in traumatic and vascular patients. This is in agreement with recent work on sedated and unsedated patients with DoC showing that among seven rs-fMRI networks the visual network was the most similar among controls and unsedated patients [14].

Disease duration had a weak but significant impact on the global number of networks observed (ρ = −0.19, *p* < 0.05), where, as duration increased, the number of networks decreased; when diagnostic accuracy was considered in relation to the different etiologies (Table 4), LASSO method considered disease duration the only significant clinical variable to combine with imaging data (Table S2 Supplementary Material). However, disease duration had no effect when networks were analyzed individually.

Finally, we would like to discuss some methodological issues of rs-fMRI analysis in DoC. Several important choices can affect the recognizability of networks in patients. These include the global signal regression, the motion parameter regression, and the number of components in ICA. Regarding the motion parameter regression, recent findings suggest that it is not necessary to have a high number of regression parameters (e.g., 24 parameters or more) to minimize motion artifacts [54]. In addition, censoring strategies, such as despike and spike regression, significantly improved network identifiability [54,55], which is essential in DoC. Regarding the global signal regression, there is no consensus on its inclusion in rs-fMRI analyses, even in DoC. Some studies have included it [27,47], whereas others did not [25,56,57]. Moreover, global signal regression may also increase anticorrelations [58] and, when motion artifacts are present, it can introduce connectivity patterns in which connectivity increases when nodes are close to each other, and decreases as the distance between nodes decreases [54,55]. For these reasons, our analysis included the regression of outlier volumes (spike regression) and of a limited number of motion parameters (3 rotation + 3 translation + WM signal + CSF signal) without global signal regression.

With regard to the selection of ICA components, the choice of using automatic methods or a fixed number of components can further impact the identification of networks. An automated method may result in a large number of components, leading to the splitting of networks, or conversely, a low number of components could lead to an underestimation of the number of networks. The choice of a fixed number of components (e.g., 20–30) is a good compromise to identify all the neural components of interest as well as to identify the known artifactual components in these patients. In addition, this type of patient has severe neuroanatomical alterations and it is often difficult to determine whether a component is neural or vascular. To better distinguish the neural components from noise and artifact, we first performed an automatic identification and then we visually verified the components, according to Griffanti’s flowchart [34]. Finally, the choice of a fixed number of components (e.g., 20–30) is in line with other rs-fMRI studies on DoC [24,29,43,59].

### 4.4. Limitations and Future Directions

In this study, we did not consider follow-up clinical data, as it was only sparingly available. Our group of patients had non-heterogeneous scores in the CRS-R subscales, except for the visual function and this represents a limitation for rs-fMRI data correlations. In the post-anoxic etiology, there was an unbalanced proportion of VS vs. MCS (30 vs. 5), which is however typically observed in the clinical realm. For the diagnostic accuracy of the four networks subdivided by etiology, traumatic patients were associated with a low diagnostic accuracy. Future work can group traumatic patients with similar damage locations. For the rs-fMRI quantitative analysis we calculated the mean intensity of the network nodes. In the future other dimensions can be explored, for example integrating the spatial information of the connectivity map with the mean intensity. The networks included in this study are closely related to specific functions. Future studies should shed light on the comparison between accuracy provided by lower-level vs. higher-level networks.

## 5. Conclusions

The four rs-fMRI networks more related to primary functions may provide additional information on the visual, auditory and sensory-motor pathways, in some cases comparable to EP and FDG-PET techniques and in absence of clinical responses. The MVIS and LVIS networks are correlated with the clinical status, as the CRS-R subscale scores of our patients were more distributed, unlike the auditory and motor subscales.

The four rs-fMRI networks have limited diagnostic accuracy when assessed individually but, when considered altogether with MRI, they increase to a very good level (AUC = 0.80) and the model rs-fMRI map intensity+MRI rating provides additional significant information to the clinical data.

Generally, rs-fMRI analyses based on a qualitative rating or a quantitative map intensity provide convergent results. Among the clinical variables, etiology can affect diagnostic accuracy and disease duration is a significant factor in the models examined.

In conclusion, rs-fMRI is a non-invasive technique, indicated when an MRI exam is performed [8] and our data confirm that it can be clinically useful in the assessment of patients with DoC.

## Figures and Tables

**Table 1:** Summary of demographic and clinical variables. Etiology is reported as traumatic/vascular/anoxic. Age, disease duration and CRS-R scores are given as median (range). Abbreviation: N = number of patients; VS/USW = vegetative state/unresponsive wakefulness state; MCS = minimally conscious state; SD = severe disability.

	N	Etiology	Age, Year	Sex, M/F	Disease Duration, mo	CRS-R
**VS/UWS**	65	18/17/30	52 (23–79)	47/18	26 (3–252)	6 (3–8)
**MCS**	34	12/17/5	46 (19–83)	12/22	39 (6–209)	10 (7–16)
**SD**	10	3/5/2	56 (36–67)	6/4	14 (2–41)	18 (14–22)
**All patients**	**109**	**33/39/37**	**50 (19–83)**	**65/44**	**27 (2–252)**	**7 (3–22)**

**Table 2:** Diagnostic accuracy of the five models (multivariate logistic regressions) for each single network, considering imaging and imaging + clinical variables.

	Imaging					Imaging + Clinical Variables				
Models	ACCU	Bal ACCU	AUC	L	R	ACCU	Bal ACCU	AUC	L	R
**rs-fMRI rating**										
SM	0.68	0.57	0.44	0	1	0.72	0.66	0.73	0	1
AUD	0.68	0.56	0.60	2	1	0.73	0.68	0.78	2	1
LVIS	0.72	0.63	0.65	1	1	0.67	0.61	0.72	1	1
MVIS	0.68	0.57	0.61	1	0	0.69	0.63	0.72	1	0
**rs-fMRI map intensity**										
SM	0.66	0.57	0.62	2	3	0.70	0.65	0.70	3	4
AUD	0.69	0.58	0.51	2	1	0.73	0.67	0.77	2	1
LVIS	0.68	0.53	0.47	1	1	0.71	0.64	0.69	1	2
MVIS	0.67	0.52	0.51	1	0	0.68	0.60	0.68	1	1
**MRI rating**										
SM	0.66	0.56	0.64	1	0	0.67	0.61	0.73	1	0
AUD	0.59	0.47	0.62	1	1	0.67	0.61	0.70	1	1
LVIS	0.60	0.52	0.66	1	1	0.71	0.65	0.71	1	1
MVIS	0.63	0.55	0.66	1	1	0.70	0.64	0.71	1	1
**rs-fMRI rating + MRI rating**										
SM	0.69	0.60	0.68	1	1	0.74	0.69	0.74	1	1
AUD	0.67	0.59	0.65	3	3	0.68	0.62	0.76	3	3
LVIS	0.62	0.54	0.68	2	2	0.68	0.64	0.73	2	2
MVIS	0.63	0.54	0.65	2	1	0.70	0.64	0.71	2	1
**rs-fMRI map intensity + MRI rating**										
SM	0.74	0.69	0.74	3	4	0.73	0.67	0.76	3	3
AUD	0.68	0.59	0.65	3	2	0.68	0.61	0.75	3	2
LVIS	0.61	0.53	0.66	1	2	0.70	0.63	0.72	1	3
MVIS	0.66	0.58	0.66	2	1	0.67	0.59	0.71	2	1

Balance accuracy = 0.64 and AUC = 0.71. SM: Sensorimotor; AUD: auditory; LVIS: lateral visual; MVIS: medial visual; ACCU: accuracy; Bal ACCU: balanced accuracy; AUC: area under the curve; L: number of variables for the left hemisphere; R: number of variables for the right hemisphere.

**Table 3:** Frequency distribution of CRS-R subscale function scores for the different diagnostic categories.

CRS-R Subscale	VS/UWS	MCS	SD	Tot
**Motor function**				
0	0	1	0	1
1	4	1	0	5
2	61	27	2	89
3 °	0	3	0	4
4 °	0	1	1	2
5 °	0	0	4	4
6 ^	0	1	3	4
*Spearman correlation with SM network ρ = 0.11*				
**Auditory function**				
0	4	2	0	6
1	54	15	2	71
2	7	11	1	19
3 °	0	5	1	6
4 °	0	1	6	7
*Spearman correlation with AUD network ρ = 0.14*				
**Visual function**				
0	20	1	0	21
1	45	3	0	46
2 °	0	5	1	7
3 °	0	22	0	23
4 °	0	2	4	6
5 °	0	1	5	6
*Spearman correlation with LVIS network ρ =* 0.27 **				
Spearman correlation with MVIS network ρ = 0.38 ***				

Spearman correlation is reported between the subscale function and the corresponding network measured with rs-fMRI rating. ° denotes a diagnosis of MCS; ^ denotes emergence from MCS; ** *p* < 0.01; *** *p* < 0.001.

**Table 4:** Number of patients with zero score at the CRS-R subscales, for whom rs-fMRI networks was deemed present or absent.

**CRS-R Motor Subscale = 0**		
**SM** **Network** **detection**	**absent**	1
	**present**	0
**CRS-R Auditory Subscale = 0**		
**AUD Network** **detection**	**absent**	3
	**present**	3
**CRS-R Visual Subscale = 0**		
**VL** **Network** **detection**	**absent**	15
	**present**	6
**CRS-R Visual Subscale = 0**		
**VM** **Network** **detection**	**absent**	17
	**present**	4

**Table 5:** Diagnostic accuracy of the five models (multivariate logistic regressions) for the 4 networks.

**4 Networks**	Imaging					Imaging + Clinical Variables				
	ACCU	Bal ACCU	AUC	L	R	ACCU	Bal ACCU	AUC	L	R
**rs-fMRI rating**	0.71	0.65	0.73	4	4	0.76	0.72	0.81	6	6
**rs-fMRI map intensity**	0.77	0.67	0.77	7	6	0.75	0.71	0.82	5	7
**MRI rating**	0.65	0.58	0.70	2	1	0.67	0.62	0.74	2	1
**rs-fMRI rating + MRI rating**	0.69	0.61	0.70	4	2	0.82	0.78	0.84	5	7
**rs-fMRI map intensity + MRI rating**	0.75	0.71	0.80	7	6	0.74	0.71	0.82	7	5

Considering imaging and imaging + clinical variables. Balance accuracy = 0.64 and AUC = 0.71. ACCU: accuracy; Bal ACCU: balanced accuracy; AUC: area under the curve; L: number of variables of the left hemisphere; R: number of variables of the right hemisphere.

**Table 6:** Diagnostic accuracy of the five models (multivariate logistic regressions) for the 4 networks, considering the different etiologies.

				Imaging				Imaging + Clinical Variables			
	N	ACCU	Bal ACCU	AUC	L	R	ACCU	Bal ACCU	AUC	L	R
**Traumatic**											
**rs-fMRI rating**	30	0.40	0.50	0	0	0	0.47	0.50	0	0	0
**rs-fMRI map intensity**	30	0.53	0.50	0	0	0	0.67	0.50	0	0	0
**MRI rating**	30	0.53	0.47	0.48	1	0	0.50	0.50	0	0	0
**rs-fMRI rating + MRI rating**	30	0.60	0.50	0	0	0	0.60	0.50	0.26	0	1
**rs-fMRI map intensity + MRI rating**	30	0.43	0.50	0	0	0	0.63	0.58	0.57	1	0
**Vascular**											
**rs-fMRI rating**	34	0.74	0.74	0.71	3	3	0.74	0.74	0.68	3	3
**rs-fMRI map intensity**	34	0.74	0.74	0.53	1	0	0.74	0.74	0.53	1	0
**MRI rating**	34	0.47	0	0	0	0	0.47	0	0	0	0
**rs-fMRI rating + MRI rating**	34	0.74	0.74	0.67	0	1	0.74	0.74	0.67	0	1
**rs-fMRI map intensity + MRI rating**	34	0.74	0.74	0.53	1	0	0.74	0.74	0.53	1	0
**Anoxic**											
**rs-fMRI rating**	35	0.80	0.63	0.86	2	0	0.91	0.87	0.76	2	0
**rs-fMRI map intensity**	35	0.97	0.90	0.98	1	2	0.94	0.88	0.88	1	2
**MRI rating**	35	0.83	0.65	0.86	2	0	0.83	0.65	0.86	2	0
**rs-fMRI rating + MRI rating**	35	0.80	0.63	0.75	3	0	0.80	0.63	0.75	3	0
**rs-fMRI map intensity + MRI rating**	35	0.86	0.83	0.75	3	1	0.91	0.87	0.97	3	1

Models refer to imaging and imaging + clinical variables; balance accuracy = 0.64 and AUC = 0.71. N = number of patients; ACCU: accuracy; Bal ACCU: balanced accuracy; AUC: area under the curve; L: number of variables for the left hemisphere; R: number of variables for the right hemisphere.

**Table 7:** Number of networks (with percentage) identified for VS/UWS, MCS and SD patients for the 3 etiologies and the correlation of the number of networks with CRS-R total score and disease duration (DD) measured with Spearman coefficient.

Etiology	Diagnosis	N pt	N SM (%)	N AUD (%)	N LVIS (%)	N MVIS (%)	N 4 Networks (%)	CRS-R	DD
**Traumatic**	**VS/UWS**	18	8 (44%)	7 (39%)	12 (67%)	9 (50%)	36 (50%)	0.10	−0.24
	**MCS**	12	5 (42%)	4 (33%)	9 (75%)	8 (67%)	26 (54%)		
	**SD**	3	2 (67%)	2 (67%)	2 (67%)	1 (33%)	7 (58%)		
	**Total**	33	15 (45%)	13 (39%)	23 (70%)	18 (55%)	69 (52%)		
**Vascular**	**VS/UWS**	17	9 (53%)	5 (29%)	11 (65%)	13 (76%)	38 (56%)	0.16	−0.01
	**MCS**	17	7 (41%)	7 (41%)	9 (53%)	10 (59%)	33 (49%)		
	**SD**	5	3 (60%)	2 (40%)	4 (80%)	5 (100%)	14 (70%)		
	**Total**	39	19 (49%)	14 (36%)	24 (62%)	28 (72%)	85 (54%)		
**Anoxic**	**VS/UWS**	30	5 (17%)	6 (20%)	4 (13%)	4 (13%)	19 (16%)	0.39 *	−0.25
	**MCS**	5	2 (40%)	2 (40%)	4 (80%)	4 (80%)	12 (60%)		
	**SD**	2	1 (50%)	2 (100%)	1 (50%)	2 (100%)	6 (75%)		
	**Total**	37	8 (22%)	10 (27%)	9 (24%)	10 (27%)	37 (25%)		
**Total**		109	42 (39%)	37 (34%)	56 (51%)	56 (51%)	191 (44%)	0.26 **	−0.19 *

N pt: Number of patients; SM: sensorimotor; AUD: auditory; LVIS: lateral visual; MVIS: medial visual. * *p* < 0.05; ** *p* < 0.01.

## Data Availability

The data presented in this study are available on request from the corresponding author.

## Supplementary Materials

The following supporting information can be downloaded at: https://www.mdpi.com/article/10.3390/brainsci12030355/s1, Table S1: AUC, confidence interval of AUC, sensitivity, specificity, and patients wrongly classified as VS/UWS and MCS by etiology as traumatic/vascular/anoxic of single networks reported in Table 2 using LOOCV; Table S2: AUC, confidence interval of AUC, sensitivity, specificity for each single network of five models using 10-fold CV; Table S3: Variables included by LASSO method for diagnostic accuracy of single networks reported in Table 2; Table S4: AUC, confidence interval of AUC, sensitivity, specificity, and patients wrongly classified as VS/UWS and MCS by etiology as traumatic/vascular/anoxic of 4 networks reported in Table 5 using LOOCV; Table S5: AUC, confidence interval of AUC, sensitivity, specificity for the 4 models using 10-fold CV; Table S6: Variables included by LASSO method for diagnostic accuracy of the 4 networks reported in Table 5; Table S7: AUC, confidence interval of AUC, sensitivity, specificity, and patients wrongly classified as VS/UWS reported in Table 6 using LOOCV; Table S8: AUC, confidence interval of AUC, sensitivity, specificity for the 4 models by etiology using 10-fold CV; Table S9: Variables included by LASSO method for diagnostic accuracy of the 4 networks considering the different etiologies reported in Table 6; Table S10: Clinical data are reported for all patients.

## References

[1] Luaute J., Maucort-Boulch D., Tell L., Quelard F., Sarraf T., Iwaz J., Boisson D., Fischer C. (2010). Long-term outcomes of chronic minimally conscious and vegetative states. Neurology.

[2] Steppacher S.D., Huemmer C., Schwab J.M., Tannast M., Siebenrock K.A. (2014). Surgical Hip Dislocation for Treatment of Femoroacetabular Impingement: Factors Predicting 5-Year Survivorship Hip. Clin. Orthop. Relat. Res..

[3] Estraneo A., Moretta P., Loreto V., Lanzillo B., Santoro L., Trojano L. (2010). Late Recovery after Traumatic, Anoxic, or Hemorrhagic Long-Lasting Vegetative State. Neurology.

[4] Katz D.I., Polyak M., Coughlan D., Nichols M., Roche A. (2009). Natural History of Recovery from Brain Injury after Prolonged Disorders of Consciousness: Outcome of Patients Admitted to Inpatient Rehabilitation with 1-4 Year Follow-Up. Prog. Brain Res..

[5] Leonardi M., Sattin D., Raggi A. (2013). An Italian Population Study on 600 Persons in Vegetative State and Minimally Conscious State. Brain Inj..

[6] Giacino J.T., Ashwal S., Childs N., Cranford R., Jennett B., Katz D.I., Kelly J.P., Rosenberg J.H., Whyte J., Zafonte R.D. (2002). The Minimally Conscious State: Definition and Diagnostic Criteria. Neurology.

[7] Giacino J.T., Katz D.I., Schiff N.D., Whyte J., Ashman E.J., Ashwal S., Barbano R., Hammond F.M., Laureys S., Ling G.S.F. (2018). Practice Guideline Update Recommendations Summary: Disorders of Consciousness. Neurology.

[8] Kondziella D., Bender A., Diserens K., van Erp W., Estraneo A., Formisano R., Laureys S., Naccache L., Ozturk S., Rohaut B. (2020). European Academy of Neurology Guideline on the Diagnosis of Coma and Other Disorders of Consciousness. Eur. J. Neurol..

[9] Edlow B.L., Claassen J., Schiff N.D., Greer D.M. (2021). Recovery from Disorders of Consciousness: Mechanisms, Prognosis and Emerging Therapies. Nat. Rev. Neurol..

[10] Sanz L.R.D., Thibaut A., Edlow B.L., Laureys S., Gosseries O. (2021). Update on Neuroimaging in Disorders of Consciousness. Curr. Opin. Neurol..

[11] Berlingeri M., Magnani F.G., Salvato G., Rosanova M., Bottini G. (2019). Neuroimaging Studies on Disorders of Consciousness: A Meta-Analytic Evaluation. J. Clin. Med..

[12] Fox M.D., Raichle M.E. (2007). Spontaneous Fluctuations in Brain Activity Observed with Functional Magnetic Resonance Imaging. Nat. Rev. Neurosci..

[13] Rosazza C., Minati L. (2011). Resting-State Brain Networks: Literature Review and Clinical Applications. Neurol. Sci..

[14] Kirsch M., Guldenmund P., Ali Bahri M., Demertzi A., Baquero K., Heine L., Charland-Verville V., Vanhaudenhuyse A., Bruno M.-A., Gosseries O. (2017). Sedation of Patients With Disorders of Consciousness During Neuroimaging. Anesth. Analg..

[15] Mak L.E., Minuzzi L., MacQueen G., Hall G., Kennedy S.H., Milev R. (2017). The Default Mode Network in Healthy Individuals: A Systematic Review and Meta-Analysis. Brain Connect..

[16] Biswal B., Zerrin Yetkin F., Haughton V.M., Hyde J.S. (1995). Functional Connectivity in the Motor Cortex of Resting Human Brain Using Echo-Planar Mri. Magn. Reson. Med..

[17] de Luca M., Beckmann C.F., de Stefano N., Matthews P.M., Smith S.M. (2006). FMRI Resting State Networks Define Distinct Modes of Long-Distance Interactions in the Human Brain. NeuroImage.

[18] Stevens W.D., Buckner R.L., Schacter D.L. (2010). Correlated Low-Frequency BOLD Fluctuations in the Resting Human Brain Are Modulated by Recent Experience in Category-Preferential Visual Regions. Cereb. Cortex.

[19] Lewis C.M., Baldassarre A., Committeri G., Romani G.L., Corbetta M. (2009). Learning Sculpts the Spontaneous Activity of the Resting Human Brain. Proc. Natl. Acad. Sci. USA.

[20] Deco G., Corbetta M. (2011). The Dynamical Balance of the Brain at Rest. Neuroscientist.

[21] Betti V., della Penna S., de Pasquale F., Corbetta M. (2021). Spontaneous Beta Band Rhythms in the Predictive Coding of Natural Stimuli. Neuroscientist.

[22] Boly M., Massimini M., Tononi G. (2009). Theoretical Approaches to the Diagnosis of Altered States of Consciousness. Prog. Brain Res..

[23] Vanhaudenhuyse A., Noirhomme Q., Tshibanda L.J.-F., Bruno M.-A., Boveroux P., Schnakers C., Soddu A., Perlbarg V., Ledoux D., Brichant J.-F. (2010). Default Network Connectivity Reflects the Level of Consciousness in Non-Communicative Brain-Damaged Patients. Brain.

[24] Rosazza C., Andronache A., Sattin D., Bruzzone M.G., Marotta G., Nigri A., Ferraro S., Rossi Sebastiano D., Porcu L., Bersano A. (2016). Multimodal Study of Default-Mode Network Integrity in Disorders of Consciousness. Ann. Neurol..

[25] Demertzi A., Antonopoulos G., Heine L., Voss H.U., Crone J.S., de Los Angeles C., Bahri M.A., di Perri C., Vanhaudenhuyse A., Charland-Verville V. (2015). Intrinsic Functional Connectivity Differentiates Minimally Conscious from Unresponsive Patients. Brain.

[26] Sair H.I., Hannawi Y., Li S., Kornbluth J., Demertzi A., di Perri C., Chabanne R., Jean B., Benali H., Perlbarg V. (2018). Early Functional Connectome Integrity and 1-Year Recovery in Comatose Survivors of Cardiac Arrest. Radiology.

[27] Song M., Yang Y., He J., Yang Z., Yu S., Xie Q., Xia X., Dang Y., Zhang Q., Wu X. (2018). Prognostication of Chronic Disorders of Consciousness Using Brain Functional Networks and Clinical Characteristics. Elife.

[28] Giacino J., Whyte J. (2005). The Vegetative and Minimally Conscious States. J. Head Trauma Rehabil..

[29] Demertzi A., Gómez F., Crone J.S., Vanhaudenhuyse A., Tshibanda L., Noirhomme Q., Thonnard M., Charland-Verville V., Kirsch M., Laureys S. (2014). Multiple FMRI System-Level Baseline Connectivity Is Disrupted in Patients with Consciousness Alterations. Cortex.

[30] Morozova S., Kremneva E., Sergeev D., Sinitsyn D., Legostaeva L., Iazeva E., Krotenkova M., Ryabinkina Y., Suponeva N., Piradov M. (2018). Conventional Structural Magnetic Resonance Imaging in Differentiating Chronic Disorders of Consciousness. Brain Sci..

[31] Estraneo A., Masotta O., Bartolo M., Pistoia F., Perin C., Marino S., Lucca L., Pingue V., Casanova E., Romoli A. (2021). Multi-Center Study on Overall Clinical Complexity of Patients with Prolonged Disorders of Consciousness of Different Etiologies. Brain Inj..

[32] Sohn W.S., Yoo K., Lee Y.-B., Seo S.W., Na D.L., Jeong Y. (2015). Influence of ROI Selection on Resting State Functional Connectivity: An Individualized Approach for Resting State FMRI Analysis. Front. Neurosci..

[33] Soddu A., Vanhaudenhuyse A., Bahri M.A., Bruno M.-A., Boly M., Demertzi A., Tshibanda J.-F., Phillips C., Stanziano M., Ovadia-Caro S. (2012). Identifying the Default-Mode Component in Spatial IC Analyses of Patients with Disorders of Consciousness. Hum. Brain Mapp..

[34] Griffanti L., Douaud G., Bijsterbosch J., Evangelisti S., Alfaro-Almagro F., Glasser M.F., Duff E.P., Fitzgibbon S., Westphal R., Carone D. (2017). Hand Classification of FMRI ICA Noise Components. NeuroImage.

[35] Giacino J.T., Kalmar K., Whyte J. (2004). The JFK Coma Recovery Scale-Revised: Measurement Characteristics and Diagnostic Utility11No Commercial Party Having a Direct Financial Interest in the Results of the Research Supporting This Article Has or Will Confer a Benefit upon the Authors or upon Any Organization with Which the Authors Are Associated. Arch. Phys. Med. Rehabil..

[36] Lombardi F., Gatta G., Sacco S., Muratori A., Carolei A. (2007). The Italian Version of the Coma Recovery Scale-Revised (CRS-R). Funct. Neurol..

[37] Sattin D., Minati L., Rossi D., Covelli V., Giovannetti A.M., Rosazza C., Bersano A., Nigri A., Leonardi M. (2015). The Coma Recovery Scale Modified Score. Int. J. Rehabil. Res..

[38] Rossi Sebastiano D., Panzica F., Visani E., Rotondi F., Scaioli V., Leonardi M., Sattin D., D’Incerti L., Parati E., Ferini Strambi L. (2015). Significance of Multiple Neurophysiological Measures in Patients with Chronic Disorders of Consciousness. Clin. Neurophysiol..

[39] Ferraro S., Nigri A., D’Incerti L., Rosazza C., Sattin D., Rossi Sebastiano D., Visani E., Duran D., Marotta G., Demichelis G. (2020). Preservation of Language Processing and Auditory Performance in Patients With Disorders of Consciousness: A Multimodal Assessment. Front. Neurol..

[40] Sattin D., Rossi Sebastiano D., D’Incerti L., Guido D., Marotta G., Benti R., Tirelli S., Magnani F.G., Bersano A., Duran D. (2020). Visual Behaviors in Disorders of Consciousness: Disentangling Conscious Visual Processing by a Multimodal Approach. Eur. J. Neurosci..

[41] (2017). MATLAB Version 9.2.0 (R2017a).

[42] Esposito F., Scarabino T., Hyvarinen A., Himberg J., Formisano E., Comani S., Tedeschi G., Goebel R., Seifritz E., di Salle F. (2005). Independent Component Analysis of FMRI Group Studies by Self-Organizing Clustering. NeuroImage.

[43] Andronache A., Rosazza C., Sattin D., Leonardi M., D’Incerti L., Minati L. (2013). Impact of Functional MRI Data Preprocessing Pipeline on Default-Mode Network Detectability in Patients with Disorders of Consciousness. Front. Neuroinform..

[44] Yan C., Zang Y. (2010). Dparsf: A Matlab Toolbox for “Pipeline” Data Analysis of Resting-State FMRI. Front. Syst. Neurosci..

[45] Allen E.A., Erhardt E.B., Damaraju E., Gruner W., Segall J.M., Silva R.F., Havlicek M., Rachakonda S., Fries J., Kalyanam R. (2011). A Baseline for the Multivariate Comparison of Resting-State Networks. Front. Syst. Neurosci..

[46] Rosazza C., Aquino D., D’Incerti L., Cordella R., Andronache A., Zacà D., Bruzzone M.G., Tringali G., Minati L. (2014). Preoperative Mapping of the Sensorimotor Cortex: Comparative Assessment of Task-Based and Resting-State FMRI. PLoS ONE.

[47] Aubinet C., Larroque S.K., Heine L., Martial C., Majerus S., Laureys S., di Perri C. (2018). Clinical Subcategorization of Minimally Conscious State According to Resting Functional Connectivity. Hum. Brain Mapp..

[48] Koo T.K., Li M.Y. (2016). A Guideline of Selecting and Reporting Intraclass Correlation Coefficients for Reliability Research. J. Chiropr. Med..

[49] R Core Team (2020). R: A Language and Environment for Statistical Computing.

[50] Bossuyt P.M., Reitsma J.B., Bruns D.E., Gatsonis C.A., Glasziou P.P., Irwig L., Lijmer J.G., Moher D., Rennie D., de Vet H.C.W. (2015). STARD 2015: An Updated List of Essential Items for Reporting Diagnostic Accuracy Studies. BMJ.

[51] Šimundić A.-M. (2009). Measures of Diagnostic Accuracy: Basic Definitions. EJIFCC.

[52] Silva S., de Pasquale F., Vuillaume C., Riu B., Loubinoux I., Geeraerts T., Seguin T., Bounes V., Fourcade O., Demonet J.F. (2015). Disruption of Posteromedial Large-Scale Neural Communication Predicts Recovery from Coma. Neurology.

[53] Chatelle C., Bodien Y.G., Carlowicz C., Wannez S., Charland-Verville V., Gosseries O., Laureys S., Seel R.T., Giacino J.T. (2016). Detection and Interpretation of Impossible and Improbable Coma Recovery Scale-Revised Scores. Arch. Phys. Med. Rehabil..

[54] Ciric R., Wolf D.H., Power J.D., Roalf D.R., Baum G.L., Ruparel K., Shinohara R.T., Elliott M.A., Eickhoff S.B., Davatzikos C. (2017). Benchmarking of Participant-Level Confound Regression Strategies for the Control of Motion Artifact in Studies of Functional Connectivity. NeuroImage.

[55] Satterthwaite T.D., Elliott M.A., Gerraty R.T., Ruparel K., Loughead J., Calkins M.E., Eickhoff S.B., Hakonarson H., Gur R.C., Gur R.E. (2013). An Improved Framework for Confound Regression and Filtering for Control of Motion Artifact in the Preprocessing of Resting-State Functional Connectivity Data. NeuroImage.

[56] di Perri C., Bahri M.A., Amico E., Thibaut A., Heine L., Antonopoulos G., Charland-Verville V., Wannez S., Gomez F., Hustinx R. (2016). Neural Correlates of Consciousness in Patients Who Have Emerged from a Minimally Conscious State: A Cross-Sectional Multimodal Imaging Study. Lancet Neurol..

[57] Gonzalez-Castillo J., Caballero-Gaudes C., Topolski N., Handwerker D.A., Pereira F., Bandettini P.A. (2019). Imaging the Spontaneous Flow of Thought: Distinct Periods of Cognition Contribute to Dynamic Functional Connectivity during Rest. NeuroImage.

[58] Aurich N.K., Alves Filho J.O., Marques da Silva A.M., Franco A.R. (2015). Evaluating the Reliability of Different Preprocessing Steps to Estimate Graph Theoretical Measures in Resting State FMRI Data. Front. Neurosci..

[59] Soddu A., Gómez F., Heine L., di Perri C., Bahri M.A., Voss H.U., Bruno M.A., Vanhaudenhuyse A., Phillips C., Demertzi A. (2016). Correlation between Resting State FMRI Total Neuronal Activity and PET Metabolism in Healthy Controls and Patients with Disorders of Consciousness. Brain Behav..

