# Supplementary material for: Resting-State fMRI in Chronic Patients with Disorders of Consciousness: The Role of Lower-Order Networks for Clinical Assessment
Source: Brain Sci. 2022 Mar 7;12(3):355. doi: 10.3390/brainsci12030355 (PMC8946756; doi:10.3390/brainsci12030355)
Supplement: Supplementary file 1 [file brainsci-12-00355-s001.zip › brainsci-1559839-supplementary.pdf]

| | Imaging | | | | | Imaging + clinical data | | | | |
|---|---|---|---|---|---|---|---|---|---|---|
| **Models** | **AUC (CI 95%)** | Sens | Spec | VS/UWS misclassified by etiology T/B/A | MCS misclassified by etiology T/B/A | **AUC (CI 95%)** | Sens | Spec | VS/UWS misclassified by etiology T/B/A | MCS misclassified by etiology T/B/A |
| **rs-fMRI rating** | | | | | | **rs-fMRI rating** | | | | |
| SM | 0.44 (0.49 - 0.65) | 0.91 | 0.24 | 2/2/2 | 9/13/4 | 0.73 (0.62 - 0.81) | 0.85 | 0.47 | 4/6/0 | 6/7/5 |
| AUD | 0.60 (0.51 - 0.65) | 0.94 | 0.18 | 1/0/3 | 10/15/3 | 0.78 (0.63 - 0.82) | 0.83 | 0.53 | 3/8/0 | 6/7/3 |
| LVIS | 0.65 (0.55 - 0.72) | 0.91 | 0.35 | 2/4/0 | 10/11/1 | 0.72 (0.57 - 0.76) | 0.79 | 0.44 | 5/8/1 | 7/7/5 |
| MVIS | 0.61 (0.50 - 0.65) | 0.91 | 0.24 | 1/5/0 | 8/14/4 | 0.72 (0.59 - 0.78) | 0.82 | 0.44 | 3/8/1 | 4/10/5 |
| **rs-fMRI map intensity** | | | | | | **rs-fMRI map intensity** | | | | |
| SM | 0.62 (0.51 - 0.67) | 0.85 | 0.29 | 3/4/3 | 7/12/5 | 0.70 (0.66 - 0.84) | 0.80 | 0.50 | 5/7/1 | 8/4/5 |
| AUD | 0.51 (0.51 - 0.66) | 0.92 | 0.24 | 1/0/4 | 10/15/1 | 0.77 (0.61 - 0.80) | 0.86 | 0.47 | 3/4/2 | 8/7/3 |
| LVIS | 0.47 (0.50 - 0.60) | 1.00 | 0.06 | 0/0/0 | 11/16/5 | 0.69 (0.59 - 0.78) | 0.85 | 0.44 | 4/6/0 | 8/6/5 |
| MVIS | 0.51 (0.49 - 0.59) | 0.99 | 0.06 | 0/1/0 | 11/16/5 | 0.68 (0.57 - 0.75) | 0.85 | 0.35 | 3/7/0 | 7/10/5 |
| **MRI rating** | | | | | | **MRI rating** | | | | |
| SM | 0.64 (0.47 - 0.64) | 0.88 | 0.24 | 4/3/1 | 8/14/4 | 0.73 (0.66 - 0.84) | 0.79 | 0.44 | 5/8/1 | 6/8/5 |
| AUD | 0.62 (0.45 - 0.60) | 0.83 | 0.12 | 4/3/4 | 11/15/4 | 0.70 (0.58 - 0.77) | 0.79 | 0.44 | 4/9/1 | 7/8/4 |
| LVIS | 0.66 (0.51 - 0.69) | 0.77 | 0.27 | 5/6/4 | 9/12/4 | 0.71 (0.61 - 0.80) | 0.83 | 0.47 | 3/7/1 | 7/6/5 |
| MVIS | 0.66 (0.48 - 0.66) | 0.80 | 0.29 | 6/5/2 | 9/11/4 | 0.71 (0.59 - 0.78) | 0.83 | 0.44 | 4/6/1 | 7/7/5 |
| **rs-fMRI rating + MRI rating** | | | | | | **rs-fMRI rating + MRI rating** | | | | |
| SM | 0.68 (0.51 - 0.69) | 0.88 | 0.32 | 5/1/2 | 7/12/4 | 0.74 (0.61 - 0.80) | 0.85 | 0.53 | 2/7/1 | 6/6/4 |
| AUD | 0.65 (0.58 - 0.77) | 0.83 | 0.35 | 4/4/3 | 9/11/2 | 0.76 (0.61 - 0.80) | 0.80 | 0.44 | 5/8/0 | 7/9/3 |
| LVIS | 0.68 (0.50 - 0.69) | 0.79 | 0.29 | 5/7/2 | 8/14/2 | 0.73 (0.58 - 0.77) | 0.80 | 0.47 | 4/8/1 | 7/7/4 |
| MVIS | 0.65 (0.47 - 0.64) | 0.82 | 0.27 | 5/6/1 | 10/11/4 | 0.71 (0.63 - 0.82) | 0.82 | 0.47 | 3/7/2 | 7/8/3 |
| **rs-fMRI map intensity + MRI rating** | | | | | | **rs-fMRI map intensity + MRI rating** | | | | |
| SM | 0.74 (0.63 - 0.81) | 0.85 | 0.53 | 6/3/1 | 5/8/3 | 0.76 (0.64 - 0.82) | 0.85 | 0.50 | 3/6/1 | 7/6/4 |
| AUD | 0.65 (0.56 - 0.72) | 0.88 | 0.29 | 3/1/4 | 11/12/1 | 0.75 (0.62 - 0.81) | 0.82 | 0.41 | 5/6/1 | 8/9/3 |
| LVIS | 0.66 (0.47 - 0.64) | 0.79 | 0.27 | 5/5/4 | 9/13/3 | 0.72 (0.61 - 0.79) | 0.85 | 0.41 | 5/4/1 | 8/7/5 |
| MVIS | 0.66 (0.52 - 0.71) | 0.82 | 0.35 | 5/7/0 | 8/11/3 | 0.71 (0.59 - 0.77) | 0.85 | 0.32 | 3/6/1 | 10/9/4 |

**Supplementary Table S1**: AUC, confidence interval of AUC, sensitivity, specificity, and patients wrongly classified as VS/UWS and MCS by etiology as traumatic/vascular/anoxic of single networks reported in Table 2 using LOOCV.

| | Imaging | | | Imaging + clinical data | | |
|---|---|---|---|---|---|---|
| **Models** | **AUC (CI 95%)** | Sens | Spec | **AUC (CI 95%)** | Sens | Spec |
| | **rs-fMRI rating** | | | **rs-fMRI rating** | | |
| SM | 0.64 (0.50 - 0.65) | 0.91 | 0.23 | 0.71 (0.62 - 0.80) | 0.86 | 0.44 |
| AUD | 0.69 (0.51 - 0.65) | 0.94 | 0.20 | 0.81 (0.63 - 0.81) | 0.85 | 0.58 |
| LVIS | 0.65 (0.54 - 0.72) | 0.85 | 0.39 | 0.71 (0.57 - 0.76) | 0.79 | 0.44 |
| MVIS | 0.69 (0.50 - 0.65) | 0.85 | 0.29 | 0.76 (0.60 - 0.78) | 0.81 | 0.44 |
| | **rs-fMRI map intensity** | | | **rs-fMRI map intensity** | | |
| SM | 0.62 (0.51 - 0.68) | 0.85 | 0.28 | 0.67 (0.65 - 0.84) | 0.82 | 0.53 |
| AUD | 0.70 (0.51 - 0.66) | 0.92 | 0.28 | 0.81 (0.60 - 0.79) | 0.83 | 0.55 |
| LVIS | 0.54 (0.50 - 0.60) | 1.00 | 0.06 | 0.73 (0.59 - 0.78) | 0.83 | 0.43 |
| MVIS | 0.56 (0.49 - 0.59) | 0.99 | 0.06 | 0.72 (0.57 - 0.75) | 0.86 | 0.45 |
| | **MRI rating** | | | **MRI rating** | | |
| SM | 0.75 (0.48 - 0.64) | 0.85 | 0.31 | 0.77 (0.66 - 0.83) | 0.79 | 0.46 |
| AUD | 0.65 (0.45 - 0.61) | 0.83 | 0.18 | 0.72 (0.58 - 0.76) | 0.82 | 0.41 |
| LVIS | 0.68 (0.52 - 0.69) | 0.81 | 0.30 | 0.72 (0.61 - 0.81) | 0.84 | 0.48 |
| MVIS | 0.69 (0.48 - 0.66) | 0.81 | 0.32 | 0.69 (0.59 - 0.78) | 0.81 | 0.41 |
| | **rs-fMRI rating + MRI rating** | | | **rs-fMRI rating + MRI rating** | | |
| SM | 0.70 (0.51 - 0.69) | 0.86 | 0.28 | 0.75 (0.61 - 0.80) | 0.83 | 0.46 |
| AUD | 0.66 (0.58 - 0.77) | 0.85 | 0.38 | 0.76 (0.61 - 0.80) | 0.79 | 0.41 |
| LVIS | 0.67 (0.50 - 0.68) | 0.78 | 0.38 | 0.73 (0.58 - 0.76) | 0.81 | 0.44 |
| MVIS | 0.65 (0.47 - 0.65) | 0.83 | 0.28 | 0.73 (0.63 - 0.82) | 0.83 | 0.45 |
| | **rs-fMRI map intensity + MRI rating** | | | **rs-fMRI map intensity + MRI rating** | | |
| SM | 0.75 (0.64 - 0.81) | 0.83 | 0.54 | 0.79 (0.64 - 0.83) | 0.86 | 0.53 |
| AUD | 0.68 (0.56 - 0.72) | 0.89 | 0.24 | 0.73 (0.63 - 0.81) | 0.79 | 0.44 |
| LVIS | 0.72 (0.46 - 0.64) | 0.82 | 0.33 | 0.71 (0.62 - 0.80) | 0.85 | 0.41 |
| MVIS | 0.65 (0.53 - 0.70) | 0.82 | 0.32 | 0.73 (0.58 - 0.76) | 0.87 | 0.40 |

**Supplementary Table S2**: AUC, confidence interval of AUC, sensitivity, specificity for each single network of five models using 10-fold CV.

| | Imaging | Imaging + clinical variables |
|---|---|---|
| Models | Selected variables | Selected variables |
| **rs-fMRI rating** | | |
| SM | ICA SM R | Imaging + clinical variables |
| AUD | ICA AUD R; ICA AUD L; Seed AUD L | Imaging + clinical variables |
| LVIS | ICA VL R; Seed LVIS L | Imaging + clinical variables |
| MVIS | Seed MVIS L | Imaging + clinical variables |
| **rs-fMRI map intensity** | | |
| SM | Seed SM R; Seed SMA L; Seed SMA R; ICA SM L; ICA SM R | Imaging + ICA SMA L + ICA SMA R + clinical variables |
| AUD | Seed AUD L; ICA AUD L; ICA AUD R | Imaging + clinical variables |
| LVIS | Seed LVIS R; ICA LVIS L | Imaging + ICA VL R + clinical variables |
| MVIS | Seed MVIS L | Imaging + Seed VM R + clinical variables |
| **MRI rating** | | |
| SM | SM L | Imaging + clinical variables |
| AUD | H L; H R | Imaging + clinical variables |
| LVIS | OccFus L; OccFus R | Imaging + clinical variables |
| MVIS | LingCalc L; LingCalc R | Imaging + clinical variables |
| **rs-fMRI rating + MRI rating** | | |
| SM | SM L; ICA SM R | Imaging + clinical variables |
| AUD | H L; H R; ICA AUD R; ICA AUD L; Seed AUD R; Seed AUD L | Imaging + clinical variables |
| LVIS | OccFus L; OccFus R; ICA LVIS R; Seed LVIS L | Imaging + clinical variables |
| MVIS | LingCalc L; LingCalc R; Seed MVIS L | Imaging + clinical variables |
| **rs-fMRI map intensity + MRI rating** | | |
| SM | SM L; SM R; Seed SM R; Seed SMA L; Seed SMA R; ICA SM L; ICA SM R | Imaging (no ICA SM R) + clinical variables |
| AUD | H L; H R; Seed AUD L; ICA AUD L; ICA AUD R | Imaging + clinical variables |
| LVIS | OccFus L; OccFus R; Seed LVIS R | Imaging + ICA VL R + clinical variables |
| MVIS | LingCalc L; LingCalc R; Seed MVIS L | Imaging + clinical variables (No Etio1) |

**Supplementary Table S3**: Variables included by LASSO method for diagnostic accuracy of single networks reported in Table 2. In the imaging + clinical variables models (latter column), adding the clinical variables leaves the imaging variables (fMRI rating and sMRI rating) included in the models. Clinical variables are disease duration, age, and etiology (divided into 2 dummy variables that represent the 3 etiology classes). SM: Sensori-motor; AUD: Auditory; LVIS: Lateral Visual; MVIS: Medial Visual; L: left hemisphere; R: right hemisphere; ICA: Independent Component Analysis; Seed: seed-based analysis; SMA: Supplementary Motor Area; OccFus: Occipital fusiform; LingCalc: linguale calacarine cortex; H: Heschl's gyrus; Etio1: traumatic etiology

| 4 Networks | Imaging | | | | | Imaging + clinical data | | | | |
|---|---|---|---|---|---|---|---|---|---|---|
| **Models** | **AUC (CI 95%)** | Sens | Spec | VS/UWS misclassified by etiology T/B/A | MCS misclassified by etiology T/B/A | **AUC (CI 95%)** | Sens | Spec | VS/UWS misclassified by etiology T/B/A | MCS misclassified by etiology T/B/A |
| **rs-fMRI rating** | 0.73 (0.62 - 0.81) | 0.83 | 0.47 | 3/4/4 | 8/8/2 | 0.81 (0.76 - 0.92) | 0.85 | 0.59 | 3/6/1 | 6/6/2 |
| **rs-fMRI map intensity** | 0.77 (0.68 - 0.86) | 0.85 | 0.50 | 3/1/6 | 6/10/1 | 0.82 (0.74 - 0.90) | 0.83 | 0.59 | 4/5/2 | 6/4/4 |
| **MRI rating** | 0.70 (0.52 - 0.70) | 0.79 | 0.38 | 6/6/2 | 6/11/4 | 0.74 (0.65 - 0.83) | 0.77 | 0.47 | 4/10/1 | 8/6/4 |
| **rs-fMRI rating + MRI rating** | 0.70 (0.57 - 0.76) | 0.85 | 0.38 | 5/3/2 | 8/12/1 | 0.84 (0.80 - 0.94) | 0.89 | 0.68 | 3/3/1 | 5/4/2 |
| **rs-fMRI map intensity + MRI rating** | 0.80 (0.73 - 0.90) | 0.83 | 0.59 | 6/3/2 | 5/8/1 | 0.82 (0.79 - 0.93) | 0.80 | 0.62 | 6/5/2 | 6/4/3 |

**Supplementary Table S4**: AUC, confidence interval of AUC, sensitivity, specificity, and patients wrongly classified as VS/UWS and MCS by etiology as traumatic/vascular/anoxic of 4 networks reported in Table 5 using LOOCV.

| 4 Networks | Imaging | | | Imaging + clinical data | | |
|---|---|---|---|---|---|---|
| **Models** | **AUC (CI 95%)** | Sens | Spec | **AUC (CI 95%)** | Sens | Spec |
| **rs-fMRI rating** | 0.73 (0.63 - 0.81) | 0.81 | 0.40 | 0.85 (0.77 - 0.92) | 0.83 | 0.58 |
| **rs-fMRI map intensity** | 0.82 (0.68 - 0.86) | 0.81 | 0.55 | 0.84 (0.73 - 0.90) | 0.83 | 0.65 |
| **MRI rating** | 0.75 (0.51 - 0.70) | 0.79 | 0.35 | 0.72 (0.64 - 0.83) | 0.80 | 0.45 |
| **rs-fMRI rating + MRI rating** | 0.73 (0.57 - 0.76) | 0.82 | 0.45 | 0.84 (0.80 - 0.94) | 0.88 | 0.68 |
| **rs-fMRI map intensity + MRI rating** | 0.81 (0.73 - 0.89) | 0.81 | 0.56 | 0.81 (0.79 - 0.93) | 0.79 | 0.63 |

**Supplementary Table S5**: AUC, confidence interval of AUC, sensitivity, specificity for the 4 models using 10-fold CV.

| Models | n | ACCU | AUC | SM | | | AUD | | | LVIS | | | MVIS | | | Hemisphere | | Clinical variables |
|---|---|---|---|---|---|---|---|---|---|---|---|---|---|---|---|---|---|---|
| **Imaging** | | | | ICA | SBA | MRI | ICA | SBA | MRI | ICA | SBA | MRI | ICA | SBA | MRI | L | R | |
| **rs-fMRI rating** | 99 | 0.71 | 0.82 | 1 | 1 | | 2 | 1 | | 1 | 1 | | | 1 | | 4 | 4 | |
| **rs-fMRI map intensity** | 99 | 0.77 | 0.88 | 3 | 3 | | 2 | | | 1 | 2 | | | 2 | | 7 | 6 | |
| **MRI rating** | 99 | 0.65 | 0.74 | | | 1 | | | | | | 1 | | | 1 | 2 | 1 | |
| **rs-fMRI rating + MRI rating** | 99 | 0.69 | 0.80 | 1 | | 1 | 1 | | | | | 1 | | 1 | 1 | 4 | 2 | |
| **rs-fMRI map intensity + MRI rating** | 99 | 0.75 | 0.91 | 2 | 3 | 1 | 1 | 1 | | | 1 | 1 | | 2 | 1 | 7 | 6 | |
| **Imaging + clinical variables** | | | | | | | | | | | | | | | | | | |
| **rs-fMRI rating** | 99 | 0.76 | 0.93 | 2 | 2 | | 2 | 1 | | 1 | 1 | | 2 | 1 | | 6 | 6 | 4 |
| **rs-fMRI map intensity** | 99 | 0.75 | 0.94 | 3 | 3 | | 2 | | | 1 | 1 | | | 2 | | 5 | 7 | 4 |
| **MRI rating** | 99 | 0.67 | 0.81 | | | 1 | | | | | | 1 | | | 1 | 2 | 1 | 3 (no Etio1) |
| **rs-fMRI rating + MRI rating** | 99 | 0.82 | 0.95 | | | 1 | | | 1 | | | | | | 1 | 5 | 7 | 4 |
| **rs-fMRI map intensity + MRI rating** | 99 | 0.74 | 0.94 | 1 | 3 | 1 | 1 | 1 | | 1 | | 1 | | 2 | 1 | 7 | 5 | 4 |

**Supplementary Table S6**: Variables included by LASSO method for diagnostic accuracy of the 4 networks reported in Table 5. Variables are reported per network (SM, AUD, LVIS and MVIS), type of data (ICA, SBA, MRI) and hemisphere. The distribution of variables is similar for the 4 networks in the imaging and imaging+clinical variables models. SM: Sensori-motor; AUD: Auditory; LVIS: Lateral Visual; MVIS: Medial Visual; L: left hemisphere; R: right hemisphere; ICA: Independent Component Analysis; SBA: seed-based analysis; Clinical variables include etiology (divided into 2 dummy variables that represent the 3 etiology classes), disease duration and age. ACCU: Accuracy; AUC: area under the curve; Etio1: traumatic etiology.

| | Imaging | | | | | Imaging + clinical data | | | | |
|---|---|---|---|---|---|---|---|---|---|---|
| | **AUC (CI 95%)** | Sens | Spec | VS/UWS misclassified | MCS misclassified | **AUC (CI 95%)** | Sens | Spec | VS/UWS misclassified | MCS misclassified |
| | **Traumatic** | | | | | **Traumatic** | | | | |
| **rs-fMRI rating** | | | | | | | | | | |
| **rs-fMRI map intensity** | | | | | | | | | | |
| **MRI rating** | 0.48 (0.4 - 0.72) | 0.78 | 0.17 | 4 | 10 | | | | | |
| **rs-fMRI rating + MRI rating** | | | | | | 0.26 (0.50 - 0.50) | 1.00 | 0.00 | 0 | 12 |
| **rs-fMRI map intensity + MRI rating** | | | | | | 0.57 (0.46 - 0.76) | 0.83 | 0.33 | 3 | 8 |
| | **Vascular** | | | | | **Vascular** | | | | |
| **rs-fMRI rating** | 0.71 (0.59 - 0.88) | 0.71 | 0.77 | 5 | 4 | 0.68 (0.59 - 0.88) | 0.71 | 0.77 | 5 | 4 |
| **rs-fMRI map intensity** | 0.53 (0.59 - 0.88) | 0.59 | 0.88 | 7 | 2 | 0.53 (0.59 - 0.88) | 0.59 | 0.88 | 7 | 2 |
| **MRI rating** | | | | | | | | | | |
| **rs-fMRI rating + MRI rating** | 0.67 (0.59 - 0.85) | 0.65 | 0.82 | 6 | 3 | 0.67 (0.59 - 0.85) | 0.65 | 0.82 | 6 | 3 |
| **rs-fMRI map intensity + MRI rating** | 0.53 (0.59 - 0.85) | 0.59 | 0.88 | 7 | 2 | 0.53 (0.59 - 0.85) | 0.59 | 0.88 | 7 | 2 |
| | **Anoxic** | | | | | **Anoxic** | | | | |
| **rs-fMRI rating** | 0.86 (0.45 - 0.88) | 0.87 | 0.40 | 4 | 3 | 0.76 (0.68 - 1.00) | 0.93 | 0.80 | 2 | 1 |
| **rs-fMRI map intensity** | 0.98 (1.00 - 1.00) | 1.00 | 0.80 | 0 | 1 | 0.88 (1.00 - 1.00) | 0.97 | 0.80 | 1 | 1 |
| **MRI rating** | 0.86 (0.48 - 0.90) | 0.90 | 0.40 | 3 | 3 | 0.86 (0.48 - 0.90) | 0.90 | 0.40 | 3 | 3 |
| **rs-fMRI rating + MRI rating** | 0.75 (0.57 - 0.98) | 0.87 | 0.40 | 4 | 3 | 0.75 (0.57 - 0.98) | 0.87 | 0.40 | 4 | 3 |
| **rs-fMRI map intensity + MRI rating** | 0.75 (0.70 - 1.00) | 0.87 | 0.80 | 4 | 1 | 0.97 (1.00 - 1.00) | 0.93 | 0.80 | 2 | 1 |

**Supplementary Table S7**. AUC, confidence interval of AUC, sensitivity, specificity, and patients wrongly classified as VS/UWS reported in Table 6 using LOOCV.

| | Imaging | | | Imaging + clinical data | | |
|---|---|---|---|---|---|---|
| | AUC (CI 95%) | Sens | Spec | AUC (CI 95%) | Sens | Spec |
| | Traumatic | | | Traumatic | | |
| **rs-fMRI rating** | | | | | | |
| **rs-fMRI map intensity** | | | | | | |
| **MRI rating** | 0.58 (0.39 - 0.72) | 0.75 | 0.05 | | | |
| **rs-fMRI rating + MRI rating** | | | | 0.60 (0.50 - 0.50) | 1.00 | 0.00 |
| **rs-fMRI map intensity + MRI rating** | | | | 0.78 (0.46 - 0.76) | 0.85 | 0.30 |
| | Vascular | | | Vascular | | |
| **rs-fMRI rating** | 0.78 (0.59 - 0.88) | 0.70 | 0.75 | 0.73 (0.59 - 0.88) | 0.70 | 0.70 |
| **rs-fMRI map intensity** | 0.74 (0.59 - 0.88) | 0.55 | 0.90 | 0.74 (0.59 - 0.88) | 0.55 | 0.90 |
| **MRI rating** | | | | | | |
| **rs-fMRI rating + MRI rating** | 0.74 (0.59 - 0.88) | 0.65 | 0.85 | 0.80 (0.59 - 0.88) | 0.70 | 0.85 |
| **rs-fMRI map intensity + MRI rating** | 0.73 (0.59 - 0.88) | 0.60 | 0.90 | 0.74 (0.59 - 0.88) | 0.65 | 0.90 |
| | Anoxic | | | Anoxic | | |
| **rs-fMRI rating** | 1.00 (0.47 - 0.88) | 0.87 | 0.40 | 0.90 (0.68 - 1.00) | 0.93 | 0.80 |
| **rs-fMRI map intensity** | 0.93 (1.00 - 1.00) | 1.00 | 0.80 | 0.93 (1.00 - 1.00) | 0.97 | 0.80 |
| **MRI rating** | 0.93 (0.48 - 0.90) | 0.90 | 0.40 | 0.93 (0.48 - 0.90) | 0.90 | 0.40 |
| **rs-fMRI rating + MRI rating** | 0.73 (0.57 - 1.00) | 0.90 | 0.40 | 0.73 (0.57 - 1.00) | 0.90 | 0.40 |
| **rs-fMRI map intensity + MRI rating** | 0.90 (0.70 - 1.00) | 0.93 | 0.80 | 0.87 (1.00 - 1.00) | 0.97 | 0.60 |

**Supplementary Table S8**: AUC, confidence interval of AUC, sensitivity, specificity for the 4 models by etiology using 10-fold CV.

| Models | Imaging | Imaging + clinical variables |
|---|---|---|
| | **Selected variables** | **Selected variables** |
| **Traumatic** | | |
| **rs-fMRI rating** | NA | NA |
| **rs-fMRI map intensity** | NA | NA |
| **MRI rating** | SM L | NA |
| **rs-fMRI rating + MRI rating** | NA | ICA AUD R |
| **rs-fMRI map intensity + MRI rating** | NA | Seed MVIS L |
| **Vascular** | | |
| **rs-fMRI rating** | ICA SM R; Seed SM R; Seed SMA L; ICA AUD L; Seed LVIS R | DD + imaging |
| **rs-fMRI map intensity** | ICA SM L | imaging |
| **MRI rating** | NA | NA |
| **rs-fMRI rating + MRI rating** | Seed LVIS R | imaging |
| **rs-fMRI map intensity + MRI rating** | ICA SM L | imaging |
| **Anoxic** | | |
| **rs-fMRI rating** | ICA AUD L; Seed LVIS L | DD + imaging |
| **rs-fMRI map intensity** | Seed AUD R; Seed MVIS R; ICA AUD L | DD + imaging |
| **MRI rating** | LingCalc L; SM L | Imaging |
| **rs-fMRI rating + MRI rating** | LingCalc L; ICA AUD L; Seed LVIS L | imaging |
| **rs-fMRI map intensity + MRI rating** | LingCalc L; SM L; Seed AUD R; ICA AUD L | DD + imaging |

**Supplementary Table S9**. Variables included by LASSO method for diagnostic accuracy of the 4 networks considering the different etiologies reported in Table 6. The selected variables included in the five models changed across etiology classes. SM: Sensori-motor; AUD: Auditory; LVIS: Lateral Visual; MVIS: Medial Visual; ICA: Independent Component Analysis; Seed: seed-based analysis; SMA: Supplementary Motor Area; LingCalc: linguale calacarine cortex; DD: disease duration; NA: not available.

| Patient | Gender | Age | Etiology | Disease duration (months) | Diagnosis | CRS-R subscale Auditory function | CRS-R subscale Visual function | CRS-R subscale Motor function | CRS-R Total score | CRS-R Modified |
|---|---|---|---|---|---|---|---|---|---|---|
| ID_005 | M | 36 | 1 | 7 | 3 | 4 | 5 | 5 | 18 | NA |
| ID_006 | M | 45 | 1 | 10 | 1 | 1 | 0 | 2 | 5 | 4.5 |
| ID_007 | M | 47 | 1 | 19 | 3 | 1 | 4 | 2 | 14 | NA |
| ID_008 | F | 44 | 1 | 62 | 2 | 1 | 3 | 2 | 11 | 31.93 |
| ID_009 | F | 40 | 2 | 42 | 1 | 1 | 1 | 2 | 7 | 6.59 |
| ID_011 | M | 57 | 3 | 16 | 1 | 1 | 0 | 2 | 5 | 4.5 |
| ID_013 | F | 55 | 2 | 23 | 1 | 1 | 1 | 2 | 6 | 5.54 |
| ID_014 | M | 46 | 3 | 55 | 1 | 1 | 1 | 2 | 8 | 6.92 |
| ID_015 | M | 65 | 1 | 56 | 1 | 1 | 1 | 1 | 6 | 4.84 |
| ID_016 | M | 60 | 2 | 70 | 2 | 2 | 3 | 2 | 11 | 24.64 |
| ID_017 | F | 40 | 3 | 44 | 2 | 2 | 3 | 2 | 11 | 24.64 |
| ID_018 | M | 43 | 3 | 57 | 1 | 1 | 0 | 2 | 6 | 5.54 |
| ID_019 | M | 52 | 1 | 48 | 1 | 1 | 0 | 1 | 5 | 4.84 |
| ID_020 | M | 34 | 1 | 40 | 1 | 1 | 0 | 2 | 6 | 4.84 |
| ID_021 | M | 50 | 3 | 26 | 1 | 1 | 1 | 1 | 6 | 4.84 |
| ID_023 | M | 77 | 1 | 30 | 1 | 1 | 1 | 2 | 7 | 5.88 |
| ID_024 | M | 67 | 2 | 7 | 3 | 4 | 5 | 5 | 22 | NA |
| ID_025 | F | 67 | 2 | 2 | 3 | 3 | 4 | 6 | 20 | NA |
| ID_026 | M | 63 | 3 | 75 | 2 | 1 | 2 | 2 | 8 | 14.21 |
| ID_027 | M | 56 | 3 | 99 | 1 | 1 | 0 | 2 | 6 | 4.84 |
| ID_028 | M | 28 | 1 | 82 | 2 | 3 | 2 | 6 | 16 | NA |
| ID_029 | F | 54 | 1 | 31 | 3 | 2 | 4 | 4 | 18 | NA |
| ID_030 | F | 49 | 3 | 41 | 1 | 1 | 1 | 2 | 8 | 6.92 |
| ID_031 | M | 49 | 3 | 41 | 1 | 1 | 1 | 2 | 6 | 5.54 |
| ID_032 | M | 53 | 3 | 7 | 1 | 1 | 0 | 2 | 5 | 4.5 |
| ID_033 | F | 39 | 2 | 41 | 2 | 2 | 4 | 2 | 10 | 30.89 |
| ID_034 | M | 56 | 3 | 98 | 1 | 1 | 0 | 2 | 7 | 5.88 |
| ID_035 | M | 59 | 2 | 76 | 2 | 1 | 3 | 3 | 11 | 39.22 |
| ID_036 | F | 63 | 2 | 37 | 2 | 1 | 2 | 2 | 9 | 15.26 |
| ID_038 | M | 55 | 3 | 33 | 1 | 1 | 1 | 2 | 7 | 5.88 |
| ID_039 | M | 22 | 1 | 10 | 2 | 1 | 2 | 3 | 7 | 21.88 |
| ID_041 | M | 56 | 1 | 13 | 1 | 1 | 1 | 2 | 6 | 5.54 |
| ID_042 | F | 56 | 1 | 25 | 1 | 1 | 0 | 2 | 6 | 4.84 |
| ID_043 | F | 46 | 1 | 41 | 1 | 1 | 1 | 2 | 7 | 5.88 |
| ID_044 | M | 66 | 3 | 15 | 3 | 4 | 5 | 2 | 18 | NA |
| ID_045 | M | 46 | 1 | 27 | 1 | 0 | 0 | 2 | 5 | 3.79 |
| ID_046 | M | 61 | 1 | 26 | 1 | 0 | 0 | 2 | 3 | 3.13 |
| ID_048 | F | 57 | 2 | 41 | 3 | 4 | 5 | 5 | 22 | NA |
| ID_049 | M | 54 | 1 | 46 | 2 | 1 | 3 | 3 | 10 | 30.89 |
| ID_050 | F | 47 | 2 | 59 | 2 | 3 | 5 | 2 | 14 | 56.94 |
| ID_051 | F | 43 | 2 | 33 | 2 | 0 | 4 | 4 | 12 | 46.52 |
| ID_052 | F | 68 | 2 | 26 | 1 | 1 | 1 | 2 | 8 | 14.21 |
| ID_053 | F | 79 | 2 | 8 | 1 | 0 | 1 | 2 | 6 | 4.84 |
| ID_054 | F | 62 | 2 | 29 | 2 | 1 | 3 | 2 | 9 | 21.51 |
| ID_055 | F | 40 | 2 | 31 | 2 | 1 | 3 | 0 | 7 | 20.47 |
| ID_056 | M | 33 | 1 | 198 | 2 | 2 | 0 | 1 | 7 | 13.17 |
| ID_058 | M | 46 | 2 | 51 | 1 | 1 | 1 | 2 | 6 | 5.54 |
| ID_059 | F | 41 | 2 | 20 | 2 | 2 | 3 | 2 | 10 | 23.59 |

| | | | | | | | | | | |
|---|---|---|---|---|---|---|---|---|---|---|
| ID_060 | F | 39 | 3 | 141 | 1 | 1 | 0 | 2 | 6 | 4.84 |
| ID_061 | F | 56 | 2 | 22 | 1 | 1 | 1 | 2 | 7 | 5.88 |
| ID_062 | M | 65 | 3 | 16 | 1 | 1 | 1 | 2 | 7 | 5.88 |
| ID_063 | M | 52 | 3 | 21 | 1 | 1 | 1 | 2 | 6 | 4.84 |
| ID_064 | F | 50 | 2 | 65 | 1 | 1 | 0 | 2 | 5 | 4.5 |
| ID_066 | M | 41 | 3 | 42 | 1 | 1 | 1 | 2 | 7 | 5.88 |
| ID_067 | F | 62 | 2 | 21 | 1 | 1 | 0 | 2 | 5 | 4.5 |
| ID_068 | M | 73 | 2 | 9 | 1 | 2 | 1 | 2 | 8 | 6.92 |
| ID_069 | M | 44 | 3 | 12 | 1 | 1 | 0 | 2 | 5 | 4.5 |
| ID_073 | M | 49 | 3 | 33 | 1 | 1 | 1 | 2 | 7 | 5.88 |
| ID_074 | M | 60 | 3 | 34 | 1 | 1 | 1 | 2 | 7 | 5.88 |
| ID_075 | M | 29 | 3 | 91 | 1 | 1 | 0 | 2 | 7 | 5.17 |
| ID_076 | M | 39 | 2 | 36 | 1 | 0 | 1 | 2 | 6 | 4.84 |
| ID_077 | M | 66 | 3 | 26 | 1 | 1 | 0 | 2 | 6 | 4.84 |
| ID_078 | M | 45 | 2 | 30 | 1 | 2 | 1 | 2 | 8 | 6.92 |
| ID_079 | M | 50 | 2 | 19 | 3 | 4 | 5 | 6 | 21 | NA |
| ID_080 | M | 49 | 1 | 16 | 1 | 1 | 1 | 2 | 7 | 5.88 |
| ID_082 | F | 47 | 3 | 209 | 2 | 3 | 3 | 2 | 11 | 31.93 |
| ID_083 | M | 44 | 2 | 48 | 1 | 1 | 1 | 1 | 6 | 4.84 |
| ID_084 | F | 83 | 2 | 119 | 2 | 0 | 3 | 2 | 8 | 13.17 |
| ID_085 | M | 52 | 3 | 9 | 1 | 1 | 0 | 2 | 6 | 4.84 |
| ID_086 | M | 33 | 3 | 16 | 1 | 1 | 1 | 2 | 6 | 4.84 |
| ID_088 | M | 60 | 1 | 43 | 1 | 1 | 1 | 2 | 6 | 5.54 |
| ID_089 | M | 25 | 1 | 12 | 1 | 1 | 1 | 2 | 6 | 5.54 |
| ID_090 | M | 44 | 3 | 70 | 1 | 1 | 1 | 2 | 7 | 5.88 |
| ID_091 | F | 25 | 1 | 12 | 2 | 1 | 1 | 2 | 7 | 13.88 |
| ID_094 | M | 38 | 1 | 41 | 2 | 2 | 3 | 2 | 12 | 24.64 |
| ID_095 | M | 76 | 2 | 15 | 1 | 1 | 1 | 2 | 8 | 22.22 |
| ID_097 | F | 71 | 2 | 51 | 2 | 2 | 3 | 2 | 10 | 23.59 |
| ID_098 | F | 53 | 2 | 9 | 1 | 1 | 1 | 2 | 5 | 4.5 |
| ID_100 | M | 59 | 2 | 24 | 2 | 3 | 3 | 2 | 12 | 32.26 |
| ID_102 | M | 30 | 1 | 17 | 1 | 1 | 1 | 2 | 6 | 5.54 |
| ID_104 | F | 58 | 1 | 29 | 2 | 1 | 3 | 2 | 9 | 22.55 |
| ID_105 | M | 37 | 3 | 12 | 3 | 4 | 2 | 5 | 17 | NA |
| ID_106 | F | 37 | 2 | 32 | 2 | 2 | 3 | 2 | 10 | 23.59 |
| ID_107 | M | 35 | 1 | 9 | 1 | 1 | 1 | 2 | 7 | 5.88 |
| ID_109 | M | 23 | 1 | 14 | 1 | 1 | 1 | 2 | 7 | 5.88 |
| ID_110 | M | 63 | 3 | 6 | 1 | 2 | 0 | 2 | 6 | 5.54 |
| ID_112 | F | 61 | 3 | 3 | 1 | 1 | 1 | 2 | 6 | 5.54 |
| ID_113 | M | 45 | 3 | 14 | 1 | 1 | 1 | 2 | 7 | 5.88 |
| ID_114 | M | 52 | 3 | 146 | 1 | 1 | 1 | 2 | 8 | 14.21 |
| ID_116 | F | 34 | 2 | 9 | 2 | 1 | 3 | 2 | 11 | 31.93 |
| ID_117 | F | 39 | 3 | 12 | 2 | 1 | 2 | 2 | 8 | 14.21 |
| ID_118 | F | 67 | 2 | 58 | 1 | 1 | 1 | 2 | 7 | 5.88 |
| ID_120 | F | 68 | 3 | 7 | 2 | 2 | 3 | 2 | 10 | 23.59 |
| ID_121 | F | 82 | 2 | 11 | 2 | 1 | 3 | 2 | 9 | 22.55 |
| ID_122 | M | 33 | 1 | 5 | 1 | 1 | 1 | 2 | 6 | 5.54 |
| ID_123 | F | 42 | 3 | 25 | 1 | 2 | 1 | 2 | 8 | 6.92 |
| ID_124 | M | 52 | 3 | 17 | 1 | 1 | 1 | 2 | 7 | 5.88 |

| | | | | | | | | | | |
|---|---|---|---|---|---|---|---|---|---|---|
| ID_126 | F | 69 | 2 | 15 | 2 | 2 | 3 | 2 | 10 | 23.59 |
| ID_128 | M | 38 | 3 | 8 | 1 | 1 | 1 | 2 | 6 | 5.54 |
| ID_129 | F | 57 | 2 | 12 | 1 | 1 | 1 | 2 | 7 | 5.88 |
| ID_130 | F | 60 | 2 | 9 | 3 | 1 | 4 | 6 | 14 | NA |
| ID_131 | F | 66 | 3 | 14 | 1 | 2 | 0 | 2 | 7 | 5.88 |
| ID_132 | M | 38 | 1 | 252 | 1 | 2 | 1 | 2 | 8 | 6.92 |
| ID_134 | F | 57 | 2 | 5 | 1 | 2 | 1 | 2 | 8 | 6.92 |
| ID_135 | M | 21 | 1 | 47 | 2 | 1 | 1 | 2 | 8 | 14.92 |
| ID_138 | M | 52 | 1 | 178 | 2 | 1 | 3 | 2 | 9 | 22.55 |
| ID_139 | F | 22 | 1 | 27 | 2 | 3 | 1 | 2 | 9 | 14.21 |
| ID_142 | F | 19 | 2 | 6 | 2 | 4 | 3 | 2 | 12 | 39.22 |
| ID_143 | M | 61 | 1 | 103 | 2 | 2 | 3 | 2 | 10 | 23.59 |

**Supplementary Table S10**. Clinical data are reported for all patients. Diagnosis: 1=Vegetative state/unresponsive wakefulness syndrome (VS/UWS); 2=Minimally-conscious state (MCS); 3=Severe disability (SD). Etiology: 1=traumatic, 2=vascular, 3=anoxic.
